# Unusual genome expansion and transcription suppression in ectomycorrhizal *Tricholoma matsutake* by insertions of transposable elements

**DOI:** 10.1371/journal.pone.0227923

**Published:** 2020-01-24

**Authors:** Byoungnam Min, Hyeokjun Yoon, Julius Park, Youn-Lee Oh, Won-Sik Kong, Jong-Guk Kim, In-Geol Choi

**Affiliations:** 1 Department of Biotechnology, College of Life Sciences and Biotechnology, Korea University, Seoul, Korea; 2 School of Life Sciences and Biotechnology, College of Natural Sciences, Kyungpook National University, Daegu, Korea; 3 Mushroom Research Division, National Institute of Horticulture and Herbal Science (NIHHS), Rural Development Administration (RDA), Eumseong, Korea; University of Muenster, GERMANY

## Abstract

Genome sequencing of *Tricholoma matsutake* revealed its unusually large size as 189.0 Mbp, which is a consequence of extraordinarily high transposable element (TE) content. We identified that 702 genes were surrounded by TEs, and 83.2% of these genes were not transcribed at any developmental stage. This observation indicated that the insertion of TEs alters the transcription of the genes neighboring these TEs. Repeat-induced point mutation, such as C to T hypermutation with a bias over “CpG” dinucleotides, was also recognized in this genome, representing a typical defense mechanism against TEs during evolution. Many transcription factor genes were activated in both the primordia and fruiting body stages, which indicates that many regulatory processes are shared during the developmental stages. Small secreted protein genes (<300 aa) were dominantly transcribed in the hyphae, where symbiotic interactions occur with the hosts. Comparative analysis with 37 Agaricomycetes genomes revealed that IstB-like domains (PF01695) were conserved across taxonomically diverse mycorrhizal genomes, where the *T*. *matsutake* genome contained four copies of this domain. Three of the IstB-like genes were overexpressed in the hyphae. Similar to other ectomycorrhizal genomes, the CAZyme gene set was reduced in *T*. *matsutake*, including losses in the glycoside hydrolase genes. The *T*. *matsutake* genome sequence provides insight into the causes and consequences of genome size inflation.

## Introduction

*Tricholoma matsutake* is an ectomycorrhizal (ECM) basidiomycete that establishes a symbiotic relationship with the roots of *Pinus densiflora*, giving it the name “pine mushroom” [1]. ECM fungi build an aggregated hyphal sheath that encases the whole root tip of its symbiotic partner and mediates the root’s external interactions with the soil [2]. This encasing or root colonization is formed through a hyphal network called the “Hartig net”, which is located inside the root cells–an anatomical pattern that is shared by the majority of ECM fungi [3,4]. The fruiting body of *T*. *matsutake* is a highly valued edible mushroom in many countries [1,5]. Unfortunately, attempts to cultivate the fruiting body have been unsuccessful, and the mechanism of mushroom development has not yet been fully understood.

Mushroom formation is proceeded by distinct developmental stages that include the vegetative hyphae stage, the dikaryotic primordia stage, and the mature fruiting body stage [6]. Various genes, including transcriptional factors [7], hydrophobins [8], and light receptors [9], have been suggested as critical genetic factors for fruiting body formation in basidiomycetes. Systematic transcriptomic surveys on fruiting body formation have been carried out for various basidiomycetes [10].

Transposable elements (TEs) play an important role in genome evolution by causing chromosomal rearrangements or by reshaping the regulatory networks [11,12]. Many ECM genomes show a high TE content, leading to comparably larger genome sizes [13], and contain more TEs than their asymbiotic relatives [14]. The effect of the presence of TEs in mushrooms is transcriptional repression, particularly when genes are surrounded by the TEs [15]. In a recent comparative genomic study of two mushroom strains, *Pleurotus ostreatus* PC15 and PC9, the genes surrounded by transposons in one strain showed strong transcriptional repression, whereas their orthologs in the other strain were normally expressed [15]. Despite the higher TE content in ECM genomes, the transcription tendency of the ECM genes affected by TEs has not been thoroughly examined.

Here, we report the genome sequence of *T*. *matsutake* and the transcriptional dynamics over three distinct developmental stages. The most distinct features of the *T*. *matsutake* genome were genome expansion by the many TEs and prevailing transcriptional suppression in all developmental stages. In addition, we performed comparative analyses on the *T*. *matsutake* and 37 Agaricomycetes genomes to identify potential gene clusters involved in symbiosis.

## Results and discussion

### Genomic summary of *T*. *matsutake*

Sequencing of the dikaryotic genomic DNA of *T*. *matsutake* generated a total length of 189.0 Mbp within 5,255 scaffolds with 111.8× sequencing coverage. We predicted 15,305 gene models using the FunGAP pipeline [16]. The predicted genes were examined for their reliability by RNA-seq, functional domains, and orthologs; thereby, the 14,528 (94.9%) genes were supported by at least one piece of evidence (Fig A in S1 File). A genome completeness test using BUSCO v3.0.2 [17] showed >99% coverage of single-copy orthologs in Basidiomycota (1,323 of 1,335 entries), validating the complete genome assembly and annotation. Because the genome was dikaryotic, we investigated how many genes were allelic by comparing two-member gene families with their relative genomes. As a result, we identified that allele genes were not frequent in the assembled genome because of its lack of two-member gene family expansion (Fig A in S1 File). We also confirmed that there was no contaminated sequence in the final assembly (Fig B in S1 File). K-mer frequency of the genomic DNA reads showed a bimodality, indicating the diploidy (Fig C in S1 File).

**Fig 1:**
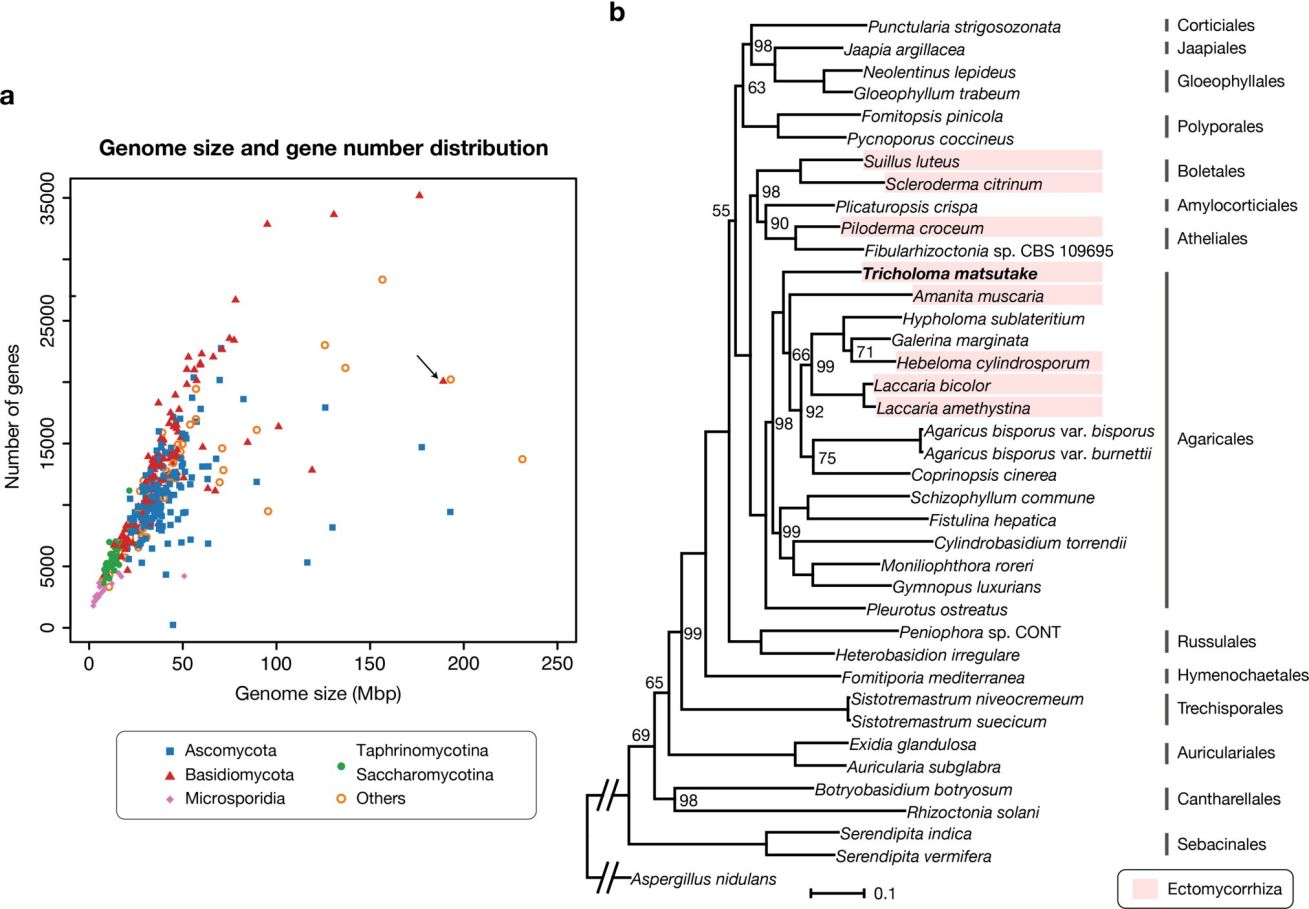
The genome sequencing of *Tricholoma matsutake*. **a.** Genome size vs. gene number of all available fungal genomes in the NCBI. As of September 2019, 5,415 fungal genome assemblies had been deposited, and 1,618 had gene predictions. We used one genome per genus to draw the plot. *Tricholoma matsutake* is indicated by the arrow. **b.** Species tree of *Tricholoma matsutake* and 37 Agaricomycetes genomes. Only bootstrap values less than 100 are marked. The scale bar that represents the mean number of amino acid substitutions per site is shown. The *Aspergillus nidulans* genome (GenBank: GCF_000149205.2) was used as an outgroup. The branch to the outgroup was shortened for visualization purposes.

As of September 2019, genome sizes of sequenced fungi deposited in the NCBI ranged from 2 Mbp to 2.1 Gbp, with an average of 31.0 Mbp (40.7 Mbp for basidiomycetes), and the *T*. *matsutake* genome had a relatively large size (Fig 1). In contrast with the size of the genome, the gene-to-genome ratio was comparatively low (81 genes per Mbp). This indicates the presence of many noncoding DNA regions (e.g., repetitive elements). Data concerning genome assembly and predicted genes are summarized in Table 1. The species tree of *T*. *matsutake* with 37 Agaricomycetes is shown in Fig 1.

**Table 1:** Genomic features of *Tricholoma matsutake*.

**Assembly statistics**	
Total contig length	144.2 Mbp
Total scaffold length	189.0 Mbp
Average base coverage	109.8×
Number of contigs	29,547
Number of scaffolds	5,255
N50 contig length	10.2 kbp
N50 scaffold length	93.4 kbp
G+C content (overall)	45.11%
G+C content (coding region)	49.57%
G+C content (non-coding region)	44.51%
Repeat elements	92.4 Mbp
**Predicted protein-coding genes**	
Predicted genes	15,305
Percent coding	8.94%
Average coding sequence size	1,104.04 nt
Gene density	80.97 genes/Mbp
Total exons	81,887
Total introns	66,582
Number of introns per gene (median)	4
Number of exons per gene (median)	4
Average exon length	206.35 nt
Average intron length	77.14 nt

### Repeat elements in the *T*. *matsutake* genome

**Fig 2:**
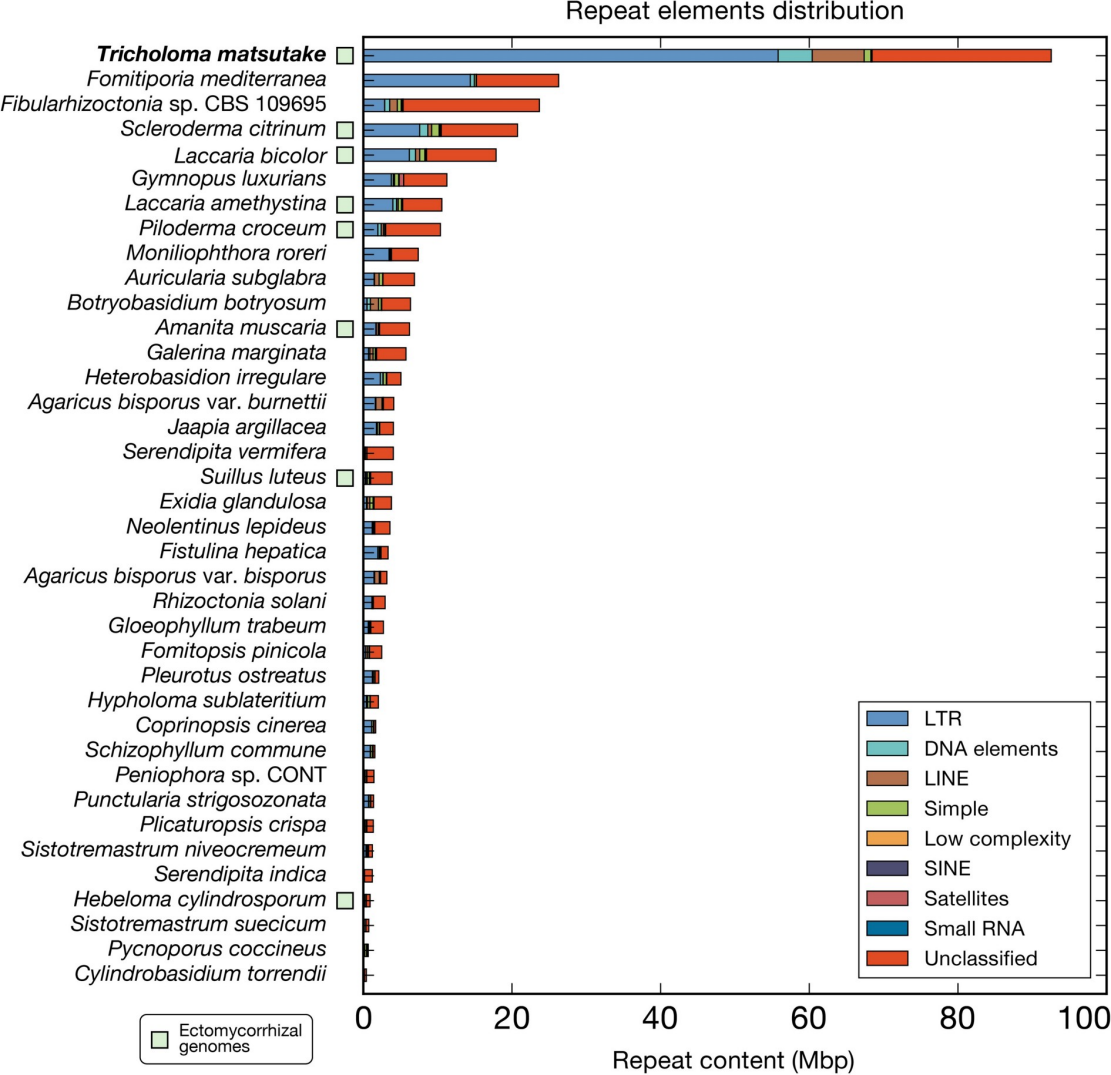
Repeat content in the *Tricholoma matsutake* and the 37 Agaricomycetes genomes. RepeatModeler and RepeatMasker (http://www.repeatmasker.org) were used sequentially to predict repeat elements in the genomes.

**Fig 3:**
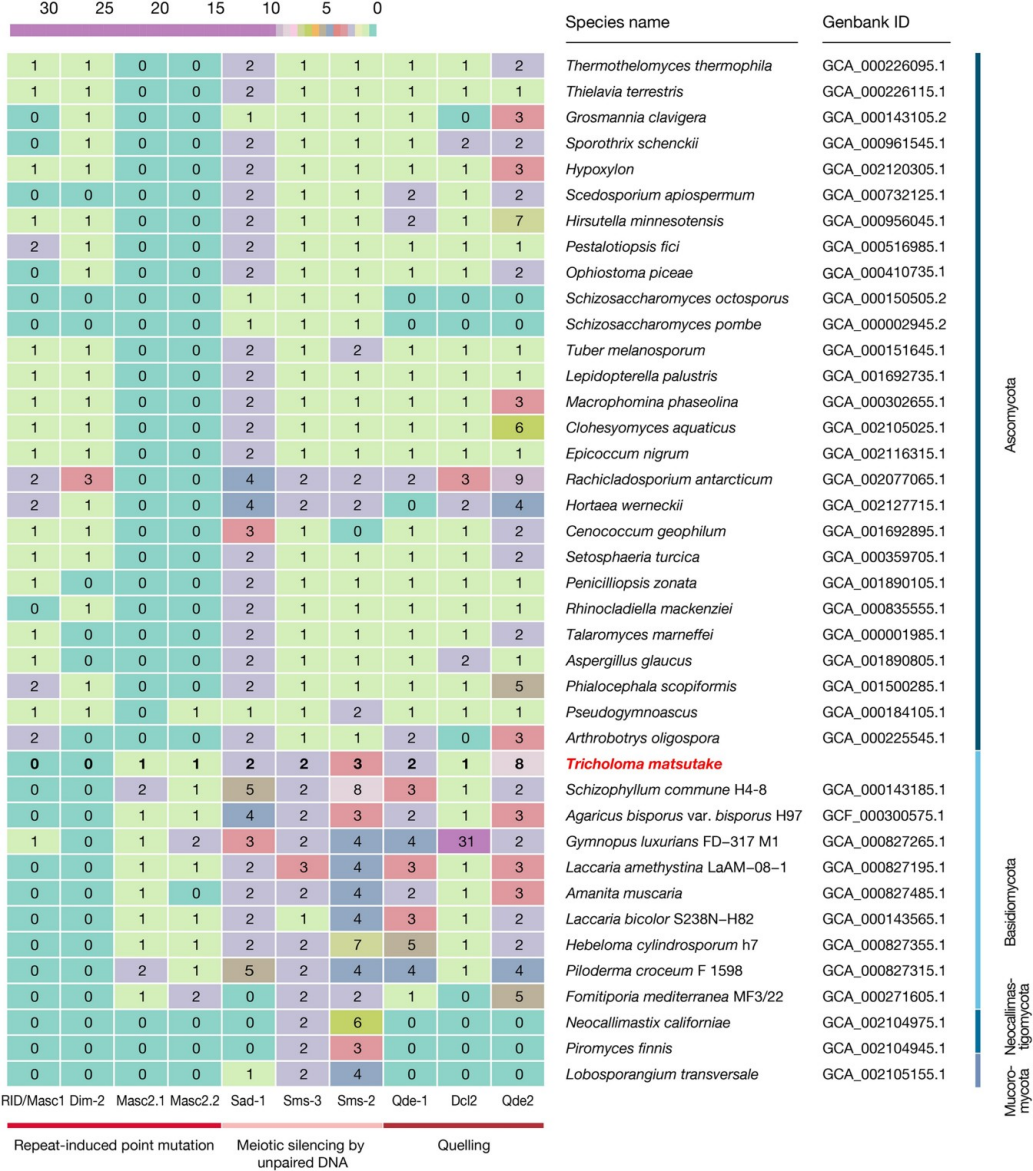
Transposable-element-silencing genes over diverse fungal genomes. The orthologs were inferred using OrthoFinder 1.0.6. Dim-2 and Masc2, Sms-3 and Dcl2, and Sms-2 and Qde2 were further differentiated from the gene trees because they belonged to the same gene families. Reference genes are listed in Table A in S2 File.

The *T*. *matsutake* genome had a high content of repeat elements, which has been suggested as a concurrent feature of ectomycorrhizal genomes [2,13,14]. The repeat elements were estimated to have a total size of 92.4 Mbp, representing 48.9% of the entire 189.0 Mbp genome (Fig 2). The major classes of repeat elements were LTR/Gypsy and LTR/Copia transposable elements, corresponding to 41 (21.9%) and 7 Mbp (3.9%) of the genome, respectively. These two frequent elements have also been observed in other basidiomycetes genomes [14,15]. Within the repeat regions, there were 15,014 complete coding sequences (containing a start codon, a stop codon, and no internal stop codon), which was equivalent to a total size of 10.9 Mbp. These were not included in the final predicted genes or further functional annotation. The majority of these sequences (14,857 of 15,014, 99.0%) were transcriptionally repressed (zero Fragments Per Kilobase of transcript per Million mapped reads (FPKMs) at all developmental stages). It is consistent with the previous report in animals that most inserted TEs are dead-on-arrival, and only a few master genes, inserted at specifically fruitful genomic locations, are transcriptionally active [18]. We presumed that meiotic silencing by the unpaired DNA and the quelling process are the potential repression mechanisms [19] because the genome included the genes responsible for those processes (*sad-1*, *sms-3*, and *sms-2* for meiotic silencing by unpaired DNA and *qde-1*, *dcl2*, and *qde2* for the quelling process; Fig 3). Another presumed mechanism is the repeat-induced point mutation (RIP). Despite a previous report that states that RIP does not exist in Agaricomycotina genomes [20], we identified the pattern of CpG hypermutations in the genome, although further studies remain to reveal whether the actual RIP process made this pattern (Fig D in S1 File). Although the genome lacked *rid/dim2* responsible for the RIP process [21] (Fig 3), its homolog, *masc2*, existed with two copies. This pattern is also frequent in other basidiomycetes genomes [22]. Experimental validation remains to be done to reveal the exact function of Masc2 in the control of the RIP process. Intergenic region length distribution indicated that many genes were located in the gene-sparse regions mainly because of the presence of enriched TEs (Fig E in S1 File).

### Genes surrounded by transposable elements are transcriptionally repressed

It was previously reported that the transcription of TE-surrounded genes is highly repressed in the fungus *Pleurotus ostreatus* [15]. There were 702 genes that were found to be surrounded by transposable elements using an ad hoc algorithm, described in the Methods section. Among these TE-surrounded genes, the transcripts of 584 genes (83.2%) were not identified at any developmental stage. This was a much higher percentage than the overall percentage of transcriptionally suppressed genes (34.4%). In an attempt to reveal that these suppressed genes were not pseudogenes or wrongly annotated genes, we investigated their homologs and found that 152 of the suppressed genes (26.0%) had paralogous genes that were not surrounded by TEs and were normally expressed in at least one developmental stage (>1 FPKM). Additionally, 290 of the suppressed genes (49.7%) had orthologous genes in at least five of the Agaricomycetes genomes. Although 89 out of 702 silenced genes were annotated with Pfam, the functional bias of silencing was not identified.

### Transcriptomic dynamics in the hyphae, primordia, and fruiting body developmental stages

**Fig 4:**
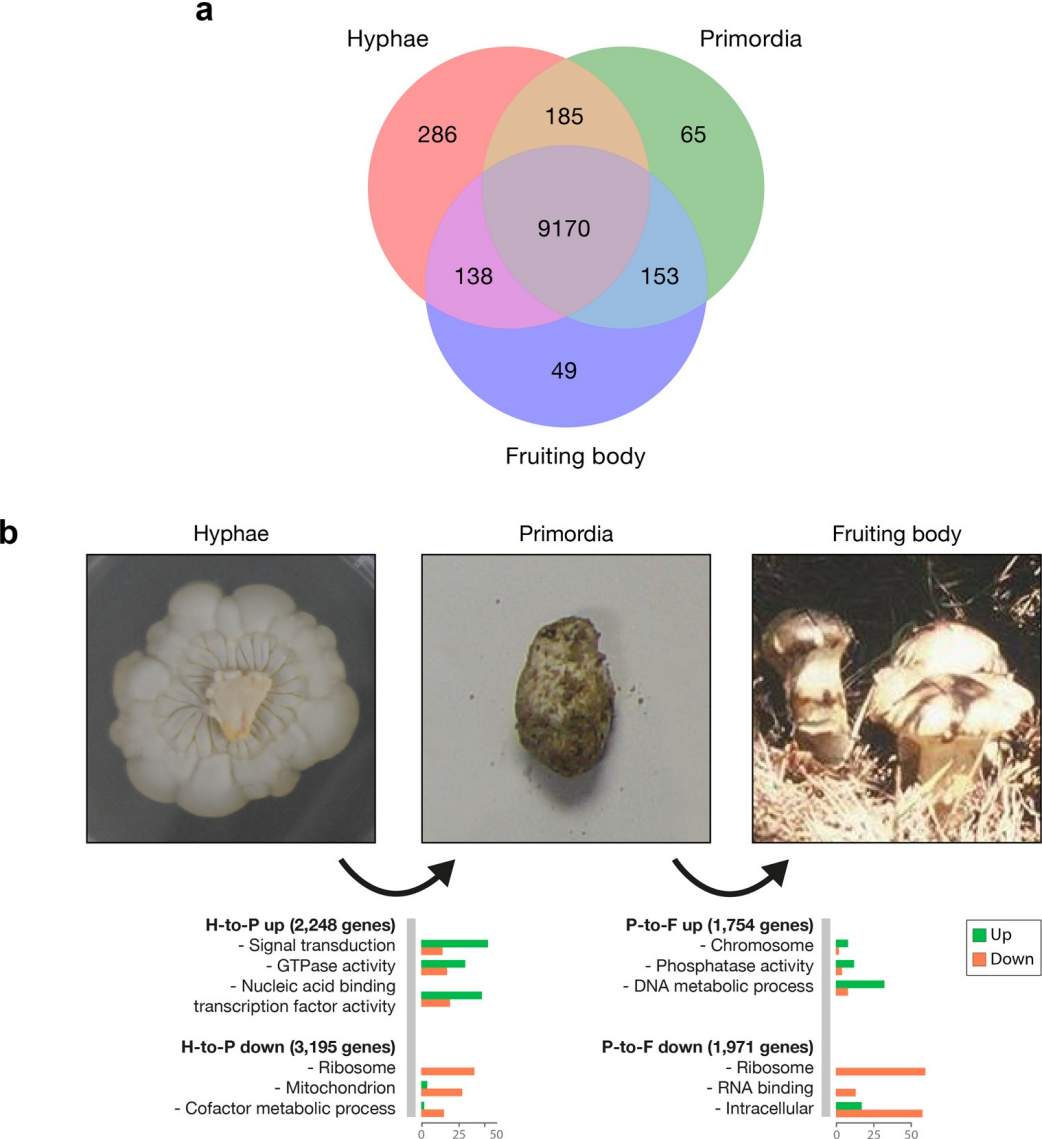
Three developmental stages of *Tricholoma matsutake*: Hyphae, primordia, and fruiting body. **a.** The Venn diagram depicts the number of expressed genes (>1 FPKM) across the three developmental stages. **b.** Upregulated and downregulated genes during development. Gene functional categorization was carried out using Gene Ontology Slim http://www.geneontology.org.

We compared transcriptomic changes between the hyphae, primordia, and fruiting body developmental stages (Fig 4). Of 15,305 predicted genes, 10,046 (65.6%) genes were transcribed in at least one condition (>1 FPKM), and 5,259 genes (34.4%) were not observed in any developmental stage. The majority of the unexpressed genes (4,976 genes, 94.6%) were annotated as hypothetical proteins that lacked known functional domains. On the contrary, 355 genes were constantly expressed during development, where the genes belonged to the top 1000 highest FPKM genes in all developmental stages. These were mostly housekeeping genes, including ribosomal proteins, heat shock proteins, cytochrome, transporters, and ATP synthases. In the hyphae, primordia, and fruiting body developmental stages, 2382, 765, and 884 genes were overexpressed over the other two stages, respectively (Data A in S3 File).

The transition from hyphae to primordia (H-to-P transition) upregulated 2248 genes and downregulated 3195 genes, and the transition from primordia to fruiting body (P-to-F transition) revealed an upregulation of 1754 genes and a downregulation of 1971 genes (Fig 4). In the H-to-P transition, the gene function related to the signal transduction, the GTPase activity, and the nucleic acid binding transcription factor activity were enriched, whereas the genes related to the ribosome, the mitochondrion, and the cofactor metabolic process were downregulated. On the other hand, in the P-to-F transition, the enriched functional categories were chromosome, phosphatase activity, and DNA metabolic process, whereas ribosome, RNA binding, and intracellular genes were suppressed (P < 0.01, estimated by Fisher’s exact test).

*Trima_09940* was the most expressed gene in the fruiting body stage (39,299 FPKM). The translated protein had a length of 158 aa and a signal sequence for secretion. In addition, this gene had a diedel domain (PF13164), which is related to the insect immune response [23]. The homologs based on sequence similarity were found in *Piloderma croceum* (ectomycorrhizal basidiomycete), *Sistotremastrum niveocremeum* (saprotrophic basidiomycete), and *Fusarium mangiferae* (plant pathogenic ascomycete). The homolog in Drosophila was also identified with 45.8% identity. Although the biological or molecular function of this gene was unclear, it is thought that it may play an essential role in fruiting body formation.

### Transcriptional regulators related to fruiting body formation: Transcription factors, light receptors, and hydrophobins

**Fig 5:**
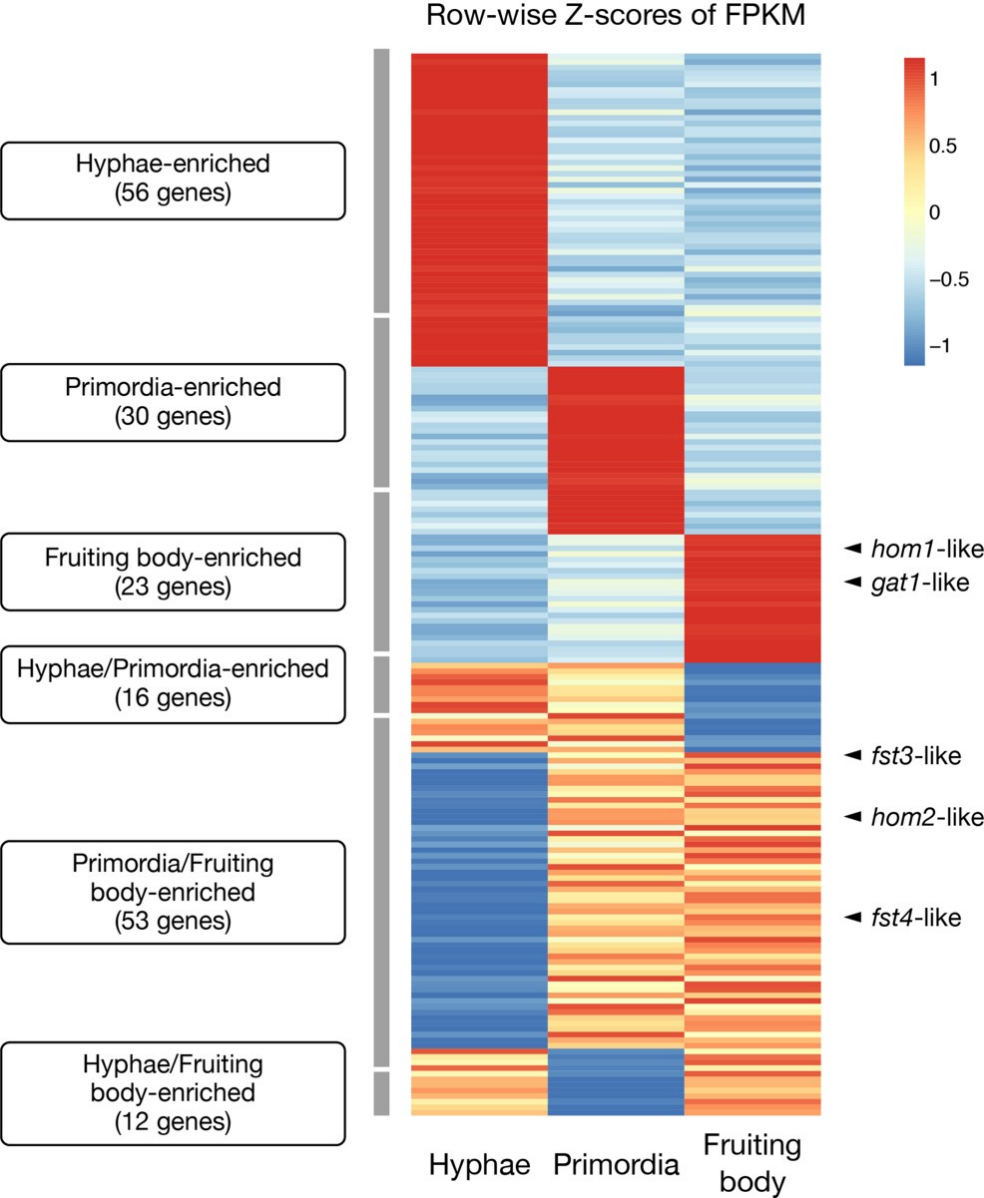
Transcription factor expressions at the three developmental stages. Differentially expressed genes were determined based on logFC (>1 or <−1), calculated by IsoEM2 and IsoDE2. When two conditions were more expressed than the other but there was no difference between them (−1 < logFC < 1), we assigned this gene as being co-overexpressed in these two conditions. Row-wise Z-scores of fragments per kilobase of transcript per million mapped reads were used.

We identified 370 transcription factor genes using a DNA-binding domain search. These included *fst4*, *fst3*, *hom1*, *hom2*, *bri1*, *gat1*, and *c2h2* homologs that play critical roles in mushroom formation [7,24] (Table B in S2 File). Although the five genes (*fst4*, *fst3*, *hom1*, *hom2*, and *gat1*) were overexpressed at the primordia and fruiting body developmental stages, as reported in previous studies, the expression levels of *bri1* and *c2h2* were not significantly changed over the three stages. Among 190 differentially expressed genes of the transcription factor (fold change > 2), 53 genes (27.9%) were overexpressed at both primordia and fruiting body developmental stages (Fig 5). This indicates that these two stages share many regulatory processes that are not shared with the hyphae stage. The transcription factors overexpressed at the primordia and fruiting body stages were classified as helix-turn-helix, basic helix-loop-helix/leucine zipper, and β-scaffold factors with minor groove contacts.

Blue-light receptor complex WC1/2 is necessary for mushroom development because its deletion prevents mushroom formation [9]. *T*. *matsutake* harbored the blue-light receptor complex WC1/2 (encoded by *Trima_13733* and *Trima_03536*). Although *wc1* gene expression was higher in the fruiting body stage, the level of the *wc2* gene was enriched in the hyphae and fruiting body (Data A in S3 File) developmental stages.

Hydrophobins have multiple biological roles that include fruiting body formation and host–fungus interaction [25]. A total of eight hydrophobin genes were annotated, which was a relatively small number compared with the other 37 Agaricomycetes genomes (Fig F in S1 File), and they ranged from 0 to 130 (average, 20). All of the hydrophobin genes were differentially expressed in the three developmental stages: four were only overexpressed at the fruiting body stage, two at the hyphae stage, and one at the primordia stage (Fig F in S1 File). A hydrophobin gene (*Trima_02415*) was overexpressed at both the hyphae and primordia stages. *Trima_15224* had the fifth highest FPKM value in the fruiting body stage among all predicted genes. This hydrophobin gene might be involved in fruiting body formation.

### Small secreted protein genes are dominantly expressed at hyphae

The small secreted protein genes of ectomycorrhizal fungi are important in symbiotic development [26]. The number of small secreted protein genes in Agaricomycetes genomes ranged from 196 to 1,053, but the ectomycorrhizal genomes had fewer small secreted protein genes, ranging from 289 to 576 (Fig G in S1 File). Among the 445 predicted small secreted protein genes, 251 genes (56.4%) were differentially expressed with at least one comparison, and 96 (21.6%) genes were not expressed (zero FPKM values) in all stages (Fig H in S1 File). Many of the differentially expressed small secreted protein genes were overexpressed at the hyphae stage (87 genes). There were 82 cysteine-rich small secreted protein genes (>3% of cysteine of translated protein sequence), such as fungal specific cysteine-rich protein (PF05730), calcium-binding protein (PF12192), peptidase inhibitor (PF03995), and various carbohydrate-binding modules. Some cysteine-rich and calcium-binding domains are involved in fungal pathogenesis [27,28].

### IstB-like domain is conserved in mycorrhizal genomes

To examine the ectomycorrhizae-specific functional domains, we performed the enrichment test over the *T*. *matsutake* and 37 Agaricomycetes genomes. Among those 21 Pfam domains enriched in the *T*. *matsutake* genome (P < 0.01, estimated by Fisher’s exact test), the IstB-like ATP-binding domain (Pfam: PF01695), which is a putative transposase [29], was highly conserved over taxonomically diverse mycorrhizal species (Fig I in S1 File). Interestingly, in the gene tree, the IstB-like genes from Basidiomycota, including *T*. *matsutake*, are closer to the ones from Mucoromycota than those from Ascomycota. This might indicate that the gene transfer between Basidiomycota and Mucoromycota occurred during evolution. It might also indicate that the gene transfer between mycorrhizae from different groups occurred during evolution. Although this domain is frequent in bacteria (http://pfam.xfam.org/family/IstB_IS21), fungal IstB-like domains were found to have low sequence similarity with the bacterial domains (<6% matched length coverages). There was lack of evidence for bacterial sequence insertion, such as the HTH-like domain in the protein sequences or the repeats on the flanks. Additionally, their homologs were conserved across the fungal kingdom. Three were overexpressed in the hyphae stage, and one was in the fruiting body stage.

We examined other outstanding functional domains that were enriched in *T*. *matsutake*: cadmium resistance transporter (PF03596), neprosin (PF03080); and carbohydrate-binding domain (PF10645, CBM52 family) (Data B in S3 File). These were mainly distributed over ascomycete genomes, and only a few basidiomycete genomes possessed them (Figs J–L in S1 File). Neprosin, a peptidase that cleaves C-terminal to proline residues under highly acidic conditions [30], is usually found in plant genomes, although few bacterial genomes have it. Among fungi, only the *Tricholoma* and *Laccaria* genomes contained neprosin (PF03080) domain. The gene tree of this domain showed the grouping between basidiomycetes and several actinobacteria genes (Fig K in S1 File). This implies that the source of this gene could be the gene transfer from actinobacteria. The CBM52 module is required for septum localization in *Schizosaccharomyces pombe* binding to β-1,3-glucan [31]. This domain (PF10645) was also contained in the *Gymnopus luxurians* and *Sistotremastrum suecicum* genomes with six and one copies, respectively. The cadmium resistance transporter (PF03596) and CBM52 gene trees were mostly consistent with their species phylogeny. Therefore, the origin of this gene family could be the common ancestor of fungi, and deletion events have occurred to make the current gene tree. Further research to verify their exact function remains to be carried out. The functional domains in the *T*. *matsutake* and other genomes are summarized in Data B in S3 File.

### CAZymes are reduced in the genome and differentially expressed during development

**Fig 6:**
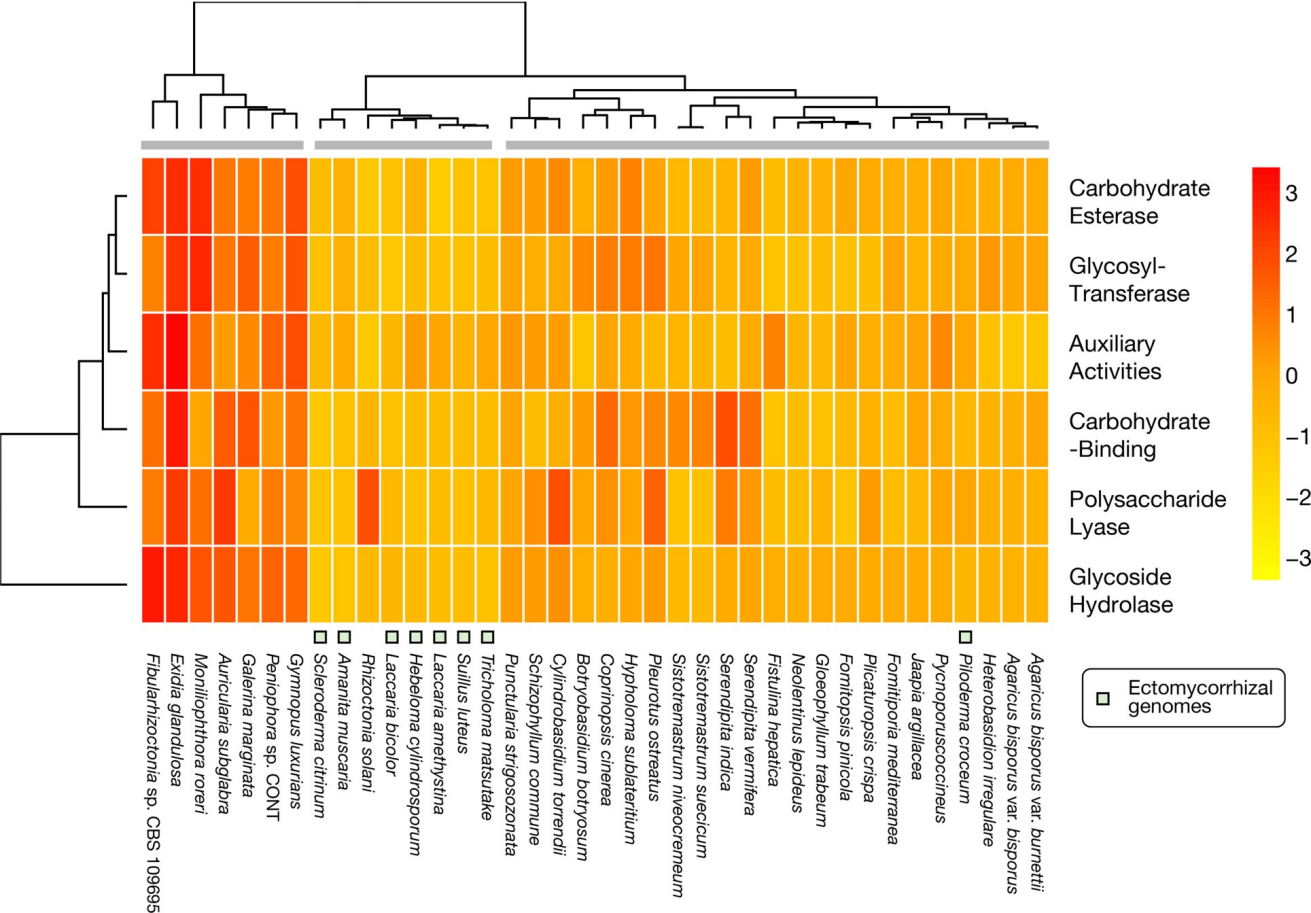
CAZyme genes in *Tricholoma matsutake* and 37 Agaricomycetes. All available CAZyme modules were counted. Scaled values based on row Z-scores were used to fill each cell.

The *T*. *matsutake* genome had a reduced number of carbohydrate-active enzymes (CAZymes) compared with other Agaricomycetes genomes. A total of 394 predicted CAZyme genes included 143 glycoside hydrolases (GHs), 33 carbohydrate-binding modules (CBMs), 90 glycosyl transferases (GTs), nine polysaccharide lyases (PLs), 59 carbohydrate esterases (CEs), and 60 auxiliary activities (AAs). Many CAZymes are involved in plant cell wall degradation. For example, some AAs, such as manganese peroxidases, versatile peroxidases, and lignin peroxidases, degrade lignin, and some GHs and CEs degrade cellulose and hemicellulose. CBMs often attach to other CAZyme domains to help them bind to target substrates. The genome had one of the lowest numbers of total CAZymes compared with the other 38 Agaricomycetes. Interestingly, ectomycorrhizal fungi, including *T*. *matsutake*, showed similar CAZyme profiles, as shown in Fig 6, except for *Piloderma croceum*. It has been reported that ectomycorrhizal basidiomycetes have lost major gene families, such as plant-cell-wall-degrading enzymes [13], which includes almost all GH families, especially the GH6 family that enzymatically degrades crystalline cellulose [13,32]. This lack of GH6 family was also observed in the *T*. *matsutake* genome. The CAZymes in the *T*. *matsutake* and other genomes are summarized in Data C in S3 File. Interestingly, we identified two CAZyme submodules that are uniquely found in the *T*. *matsutake* genome, and other ectomycorrhizal genomes lacked CBM16 and CBM52. CBM16 is common in bacteria and binds to glucomannan and kappa-carrageenan [33]. The *Trima_13517* with this module has a chitin biosynthesis protein CHS5 domain (PF16892), implying its role in cell wall biosynthesis. CBM52 binds to β-1,3-glucan [31] and is often associated with GH81 (endo-β-1,3-glucanase), although the *Trima_00904* with the CBM52 lacked any other known functional domains. Further investigation is needed to shed light on their biological functions.

**Fig 7:**
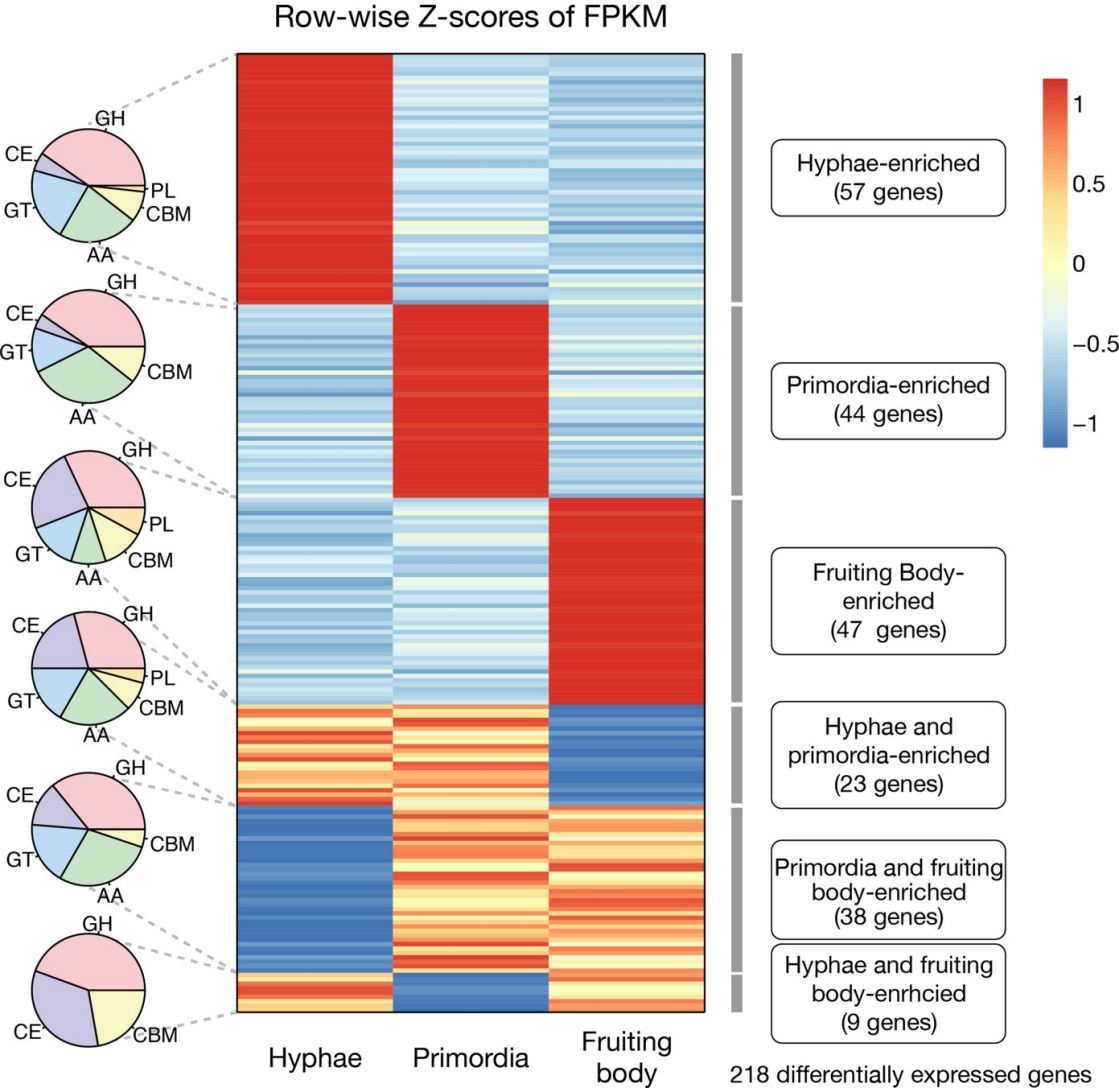
Differentially expressed CAZymes at each of the developmental stages. The pie graphs depict the number of CAZyme families for each specific expression type. Abbreviations for CAZyme families are as follows: glycoside hydrolase (GH), carbohydrate-binding module (CBM), glycosyl transferase (GT), polysaccharide lyase (PL), carbohydrate esterase (CE), and auxiliary activity (AA). Row-wise Z-scores of fragments per kilobase of transcript per million mapped reads were used.

Different numbers of CAZymes were expressed during development, and 57 at the hyphae stage, 44 at the primordia stage, and 47 at the fruiting body stage were more transcribed than in other conditions (Fig 7). Four of nine PL families were overexpressed in the fruiting body stage. Several of the GH subfamilies, such as GH5, GH17, and GH20, were only, or mostly, expressed in the fruiting body stage (4 of 15 for GH5, 2 of 2 for GH17, and 2 of 2 for GH20). GH5 has a role in the degradation of lignocellulose [32]. Interestingly, 15 of 44 (34%) primordia-activated genes were auxiliary activities (AAs), and 11 of 57 (19%) and 5 of 47 (11%) were overexpressed at the hyphae and fruiting body stages, respectively. The AA families activated at the primordia stage included AA1, AA3, AA7, and AA9. Although many AAs are involved in lignin degradation [34], their functions in primordia development are unknown.

## Methods and materials

### Strain and culture conditions

*Tricholoma matsutake* KMCC04578 at the primordia and fruiting body phases were harvested from Gachang, located near Daegu, South Korea. The dikaryotic mycelia were isolated from the gills of the fruiting bodies and cultured in potato-dextrose broth (PDB; 4 g/L potato peptone, 20 g/L glucose, and pH 5.6 ± 0.2) for 30 days at 25°C.

### Isolation of genomic DNA and total RNA

The genomic DNA was extracted from the mycelium using a cetyl trimethyl ammonium bromide (CTAB)-based fungal DNA isolation protocol [35]. The total RNA was extracted from *T*. *matsutake* (mycelium, primordium, and stipe of the fruiting body) using an RNeasy mini isolation kit (Qiagen, Valencia, CA, USA). The samples were ground to a fine powder using a mortar and pestle under liquid nitrogen. The resulting samples were homogenized with 15 mL of buffer RLT containing β-mercaptoethanol. After centrifugation for 10 min at 3,000 g, the upper phase was mixed with 15 mL of 70% EtOH, and the total RNA was isolated using an RNA Binding Spin Column under centrifugation for 5 min at 3,000 g. After two wash steps, the total RNA was extracted using DEPC-treated water. The RNA samples (A260/A280 > 1.8) were collected and subjected to further experiments.

### Genome sequencing and genome assembly

Three sequencing libraries were generated for *T*. *matsutake*: two Illumina paired-end libraries (500 and 250 bp insert sizes) and an Illumina mate pair library (5 kbp insert size) (Table C in S2 File). Raw reads were quality-controlled by trimming the low-quality bases (<30 Phred quality score) and removing short reads after trimming (<50 bp for MiSeq reads and <30 bp for HiSeq reads) using Trim Galore 0.4.4 (https://www.bioinformatics.babraham.ac.uk/projects/trim_galore/). The mitochondrial genomic reads were removed by aligning all of the reads against the reported *T*. *matsutake* mitogenome sequence (GenBank ID: JX985789.1) [36] using Bowtie2 (*-k 1—very-sensitive—end-to-end*) [37]. As a result, 1.1% of the total reads were removed.

ALLPATHS [38] was used for the assembly using the three Illumina libraries with *PLOIDY = 2* option. We identified and removed the four scaffolds derived from vector contamination obtained by the BLASTn search against the UniVec database (https://www.ncbi.nlm.nih.gov/tools/vecscreen/univec/). One scaffold of human DNA contamination was also removed. Additional sequence contaminations were examined by drawing a scatterplot of GC contents and sequence coverages using Blobology [39] with alignment of all scaffolds against the NCBI *nt* database. Thus, we corroborated that there was no additional sequence contamination in the final assembly.

### Gene prediction

FunGAP [16] was used to predict the protein-coding genes in the assembly. The genome assembly and RNA-seq reads from the hyphae stage were inputted into the program. A *Laccaria_bicolor* gene model was selected for Augustus inside the FunGAP. This generated 17,018 preliminary predicted genes. We manually removed 1,707 transposable element genes, such as retrotransposon gag protein (Pfam: PF03732) and reverse transcriptase (Pfam: PF07727) based on Pfam annotation by InterProScan 5.25–64 [40]. Targeted Pfam domains are listed in Table D in S2 File.

The remaining 15,305 genes were examined for their reliability by RNA-seq reads alignment, functional domain annotation, and ortholog search against relatives. RNA-seq reads from the three developmental stages (hyphae, primordia, and fruiting body) were aligned into the genome assembly using HISAT2 [41], and FPKM values were calculated using IsoEM2 [42]. Only >1 FPKM genes were considered as RNA-seq-supported genes. Pfam domains were annotated by InterProScan 5.25–64, and the genes containing at least one Pfam domain were considered as functional domain-supported genes. An ortholog search was performed by OrthoFinder 1.0.6 [43] using *T*. *matsutake* and 37 Agaricomycetes genomes. When a gene belonged to a gene family that contains members from more than five genomes, we considered that the gene was supported by the ortholog search. To check for genome completeness, we used BUSCO v3.0.2 [17], in which the *basidiomycota_odb9* database was used. Because the assembled genome was dikaryotic, we estimated how many genes were allelic by comparing the numbers of two-member gene families with the five Agaricales genomes. This was obtained by parsing the OrthoFinder output.

### Comparative analysis

We chose 37 Agaricomycetes genomes for comparative analyses (Table E in S2 File). In the NCBI database, as at the time of writing, there are 59 Agaricomycetes genome assemblies with predicted genes. We eliminated three incomplete genomes based on BUSCO calculations (<90% completeness). We also sampled two genomes from each order, excluding Agaricales (to which *T*. *matsutake* belongs), to reduce computing time. This yielded 38 genomes as the final targets for comparative analysis. A species tree was built using RAxML 8.1.3 [44] from the concatenated single-copy orthologs obtained by OrthoFinder 1.0.6 [43]. We used *-f a -x 12345 -p 12345 -# 100 -m PROTGAMMAWAG* options for RAxML. Mafft 7.273 [45] and Gblocks 0.91b [46] were used to align the concatenated sequences and extract the conserved regions.

### RNA-sequencing

Illumina RNA sequencing generated 108, 133, and 127 million RNA-seq reads from the hyphae, primordia, and fruiting body stages, respectively (Table F in S2 File). Trim Galore 0.4.4 (https://www.bioinformatics.babraham.ac.uk/projects/trim_galore/) was used for adapter removal, low-quality base trimming (<20 Phred score), and short-reads filtering (<40 bp). The RNA-seq reads were aligned to the genome by HISAT 2.0.5 [41]. Owing to a single replicate of the RNA-sequencing libraries, differentially expressed genes were estimated by IsoEM2 and IsoDE2, performing a bootstrapping-based approach using an accurate expectation-maximization algorithm [42]. We used the—*auto-fragment-distrib* option for IsoEM2 and the *-pval 0*.*05* (desired P value) option for IsoDE2. These programs generate a logarithm of fold change (logFC) between two conditions for each gene. When the logFC value of a certain gene is less than −1 or greater than 1, we considered that gene to be significantly differentially expressed. We classified all of the genes into seven expression patterns: hyphae-enriched, primordia-enriched, fruiting-body-enriched, hyphae- and primordia-enriched, primordia- and fruiting-body-enriched, hyphae- and fruiting-body-enriched, and not significantly different among the samples. When two conditions were more expressed than the other but there was no difference between them, we assigned this gene as being co-overexpressed in those two conditions.

To obtain upregulated or downregulated genes during development, we accounted for the differentially expressed genes in the hyphae–primordia comparison and the primordia–fruiting body comparison. The differentially expressed genes were functionally classified based on Gene Ontology terms. First, Gene Ontology terms were assigned by running InterProScan 5.25–64 [40] against the PfamA database with the—*goterms* option. Second, we assigned each Gene Ontology term to a high-level Gene Ontology term (GO slim) by running owltools (http://code.g.,oogle.com/p/owltools/) with the—*map2slim* and *—subset goslim_generic* options. Finally, Fisher’s exact enrichment test was carried out on each GO slim using Python *scipy*.*stats*.*fisher_exact* function (https://www.scipy.org/).

### Transcription factor annotation

Transcription factor genes were predicted based on the Pfam domain annotation. The transcription factor Pfam domains were obtained from the DBD database (http://www.transcriptionfactor.org) [47] in addition to the three functional domains: ARID/BRIGHT DNA-binding domain (PF01388) and fungal specific transcription factor domains (PF04082 and PF11951). The known transcription factor homologs were identified by a BLASTp search, where the best hit was selected. Their orthology relations were validated using OrthoFinder 1.0.6 [43].

### Functional domain annotation

InterProScan 5.25–64 [40] predicted the functional domains from the protein sequences of *T*. *matsutake* and 38 Agaricomycetes genomes with Pfam 31.0 [48]. The enriched and depleted functions were estimated by a Fisher’s exact test with the *scipy*.*stats*.*fisher_exact* function of Scipy Python module (https://www.scipy.org/). Four selected functions (PF01695, PF03596, PF03080, and PF10645) were BLASTp-searched against the NCBI *nr* database, and the 50 top-hit sequences were used to build the gene trees. Mafft 7.273 [45] was used for multiple genome sequence alignment with the—*maxiterate 1000—localpair* options. FastTree 2.1.10 [49] built the trees with default options.

### CAZyme annotation

Carbohydrate-related enzymes were predicted using three different tools: dbCAN HMMs 5.0 [50], a database that uses HMM profiles of known CAZyme sequences; BLASTp, a tool for searching the protein sequences against the CAZyme sequence database; and Pfam 31.0, domains annotated with CAZyme entries. All three tools were run and integrated to make a final CAZyme prediction. We assigned a gene as a CAZyme when more than two of the programs gave the same prediction on a gene.

### Small secreted protein gene prediction

Referring to previous works [51–53], we combined four extracellular protein prediction programs: SignalP, WoLF PSORT, TargetP, and ProtComp. SignalP 4.1 [54] was run with the default option, and “signal peptide = Y” and “Networks-used = SignalP-noTM” tags were used to obtain the signal peptide-containing protein sequences. WoLF PSORT [55] was used with “OrganismType = fungi,” and the most voted localization was used for each protein. TargetP 1.1 [56] was used with -N option for using non-plant networks, and “Loc = S” was used to obtain the secreted proteins. ProtComp v9 (http://www.softberry.com/berry.phtml) was used with *-NODB -NOOL* options, and “Integral Prediction of protein location” was used for assigning the protein locations. Four programs predicted 780, 1344, 2173, and 823 proteins as secreted, respectively (Fig G in S1 File). Only 78 proteins were predicted as secreted by all programs. The genes predicted by at least three programs (589 proteins) were considered as preliminary secreted protein. We excluded transmembrane, endoplasmic reticulum, and glycophosphatidylinositol-anchored proteins from the candidates with TMHMM, PS_SCAN, and GPI-SOM programs. TMHMM 2.0 [57] was used with default options, and when a transmembrane helix was located within 70 aa of the N-terminal and other helixes were not identified, we considered this as a non-transmembrane protein, with reference to previous work [58]. PS_SCAN 1.86 [59] was used to scan the endoplasmic-reticulum-targeting proteins (PROSITE: PS00014). GPI-SOM 1.5 [60] was used with default options. This resulted in 1788 transmembrane proteins, 16 endoplasmic-reticulum-targeting proteins, and 1718 glycophosphatidylinositol-anchored proteins. By the removal of these proteins, we obtained 455 proteins as secreted proteins in the *T*. *matsutake* genome.

### Repeat elements analysis

RepeatModeler and RepeatMasker (http://www.repeatmasker.org) were used sequentially to predict repeat elements in the genomes. RepeatModeler produced 2375 consensus repeat sequences with an average of 951 bp, including 289 LTR, 130 DNA, and 39 LINE elements (Table G in S2 File). Classified repeat sequences produced by RepeatModeler were used as a library for RepeatMasker.

The protein-coding sequences within repeat elements were predicted by running Braker1 [61] on the unmasked assembly. The evidence of repeat-induced point mutations was calculated by following the Amselem et al.’s (2015) method [22]. Briefly, repeat sequences were extracted into a FASTA file using *rmOut2Fasta*.*pl* script within the RepeatMasker package. We split the sequences so that one FASTA file would contain one repeat family sequence. C-to-T hypermutation of specific dinucleotides was calculated using Mafft-7.273 [45] and RIPCAL 2.0 [62]. Finally, the dinucleotide biases were calculated by counting the repeat elements with >2 transition/transversion ratio and where more than one-third of the sequences had a dinucleotide hypermutations bias. We considered genes as TE-surrounded when a gene had repeat elements at both upstream and downstream within a distance of 1000 bp. Only >400 bp repeat elements were accounted for because there were so many short fragments (139,210 elements).

The genes responsible for genome defense against TEs were identified using gene family and gene tree analyses. We targeted three mechanisms, including repeat-induced point mutation, meiotic silencing by unpaired DNA, and quelling, and nine reference genes related to these mechanisms were used to find their orthologs in the proteome of *T*. *matsutake* and the other genomes (Table A in S2 File). We identified the gene families of BLASTp top hits against the reference genes. The genes of Dim-2 and Masc2, Sms-3 and Dcl2, and Sms-2 and Qde2 belonged to the same gene family. Therefore, we additionally constructed gene trees to distinguish them, and Mafft-7.273 [45] and FastTree [49] were used to build the gene trees.

## Conclusion

This study aimed to explore the genome composition of ectomycorrhizal *Tricholoma matsutake*. The repetitive insertions of the transposable elements made this species harbor a remarkably large genome size. These inserted TEs are occasionally involved in transcriptional suppression of the nearby protein-encoding genes. We identified the evidence of genome defense against TEs by C to T hypermutation with a bias over “CpG” dinucleotides. Developmental transcriptomic dynamics revealed that many transcriptional factors are expressed in the primordia and fruiting body stages, and small secreted proteins are expressed in the hyphae stage. The genome contained less carbohydrate-active enzymes than other ectomycorrhizal fungi. These results will help in understanding how *Tricholoma matsutake* has developed and maintained its lifestyle.

## Data availability

This Whole Genome Shotgun project has been deposited at DDBJ/ENA/GenBank under the accession number PKSN00000000. The version described in this paper is version PKSN02000000. The MycoBank ID of this species is 307044. The data used in this study are available at dx.doi.org/10.6084/m9.figshare.11301098. The authors declare that all other data supporting the findings of this study are available within the article and its Supplementary Information files or are available from the corresponding authors upon request.

## Supporting information

S1 File Supporting Figures. Figs A–L.
(PDF)

S2 File Supporting Tables. Tables A–G.
(PDF)

S3 File Supporting Data. Data A–C.
(XLSX)
