# Correction: Unusual genome expansion and transcription suppression in ectomycorrhizal *Tricholoma matsutake* by insertions of transposable elements

**DOI:** 10.1371/journal.pone.0229406

**Published:** 2020-02-13

**Authors:** The PLOS ONE Staff

## Notice of republication

Incorrect versions of Figs 2 and 3 were published in error. The publisher apologizes for these errors. This article was republished on February 4, 2020 to correct for these errors. Please download this article again to view the correct version.
