# Supplementary material for: Unusual genome expansion and transcription suppression in ectomycorrhizal Tricholoma matsutake by insertions of transposable elements
Source: PLoS One. 2020 Jan 24;15(1):e0227923. doi: 10.1371/journal.pone.0227923 (PMC6980582; doi:10.1371/journal.pone.0227923)
Supplement: S1 File — Figs A–L. (PDF) [file pone.0227923.s001.pdf]

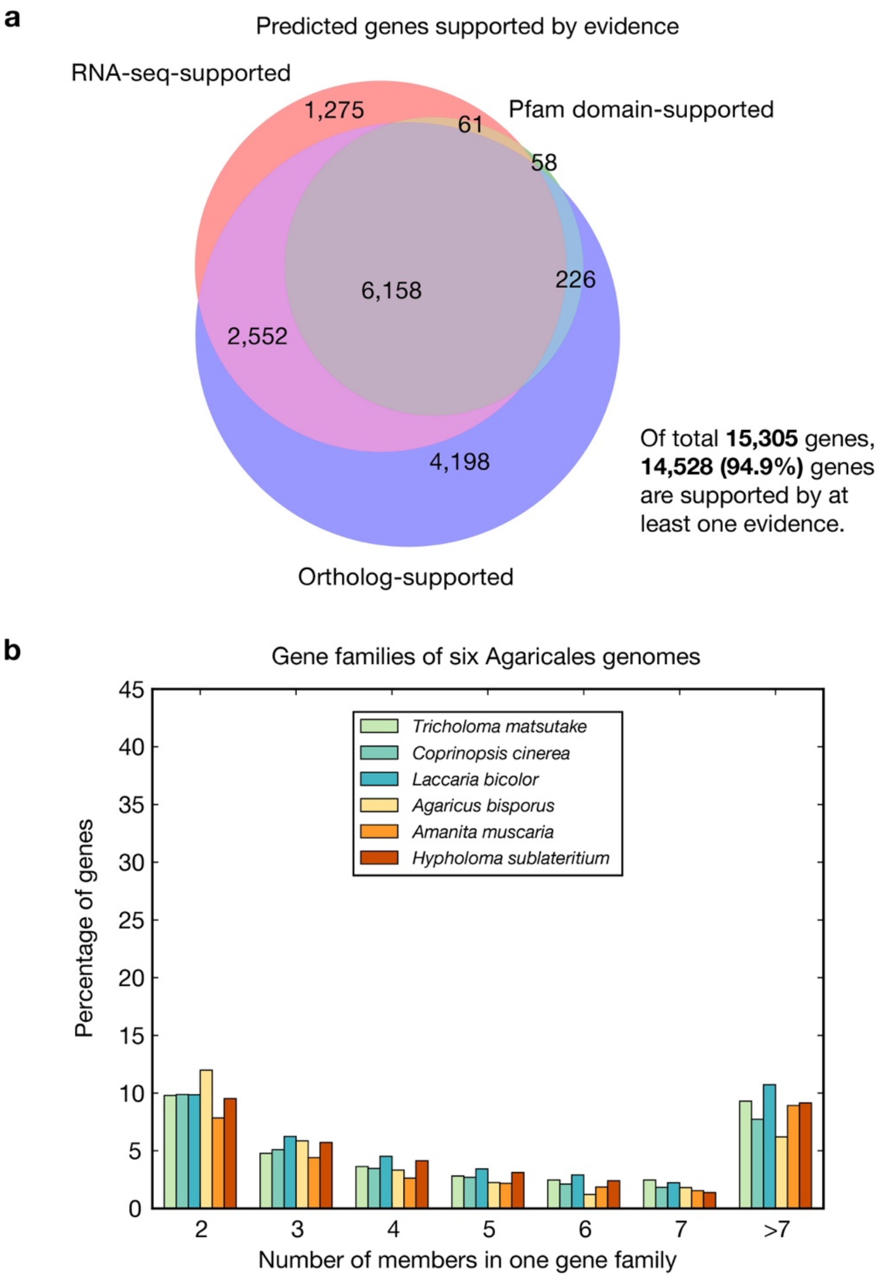

Fig A | Gene prediction quality assessment and allele genes estimation. a. The predicted genes were examined for their reliability using RNA-seq, Pfam domains, and orthologs within 37 Agaricomycetes genomes. For RNA-seq, we accounted for the genes with >1 FPKM in at least one development stage. When a gene family includes members from more than five genomes, we considered those members are reliable (not pseudogene). **b.** The frequencies of gene members in a gene family.

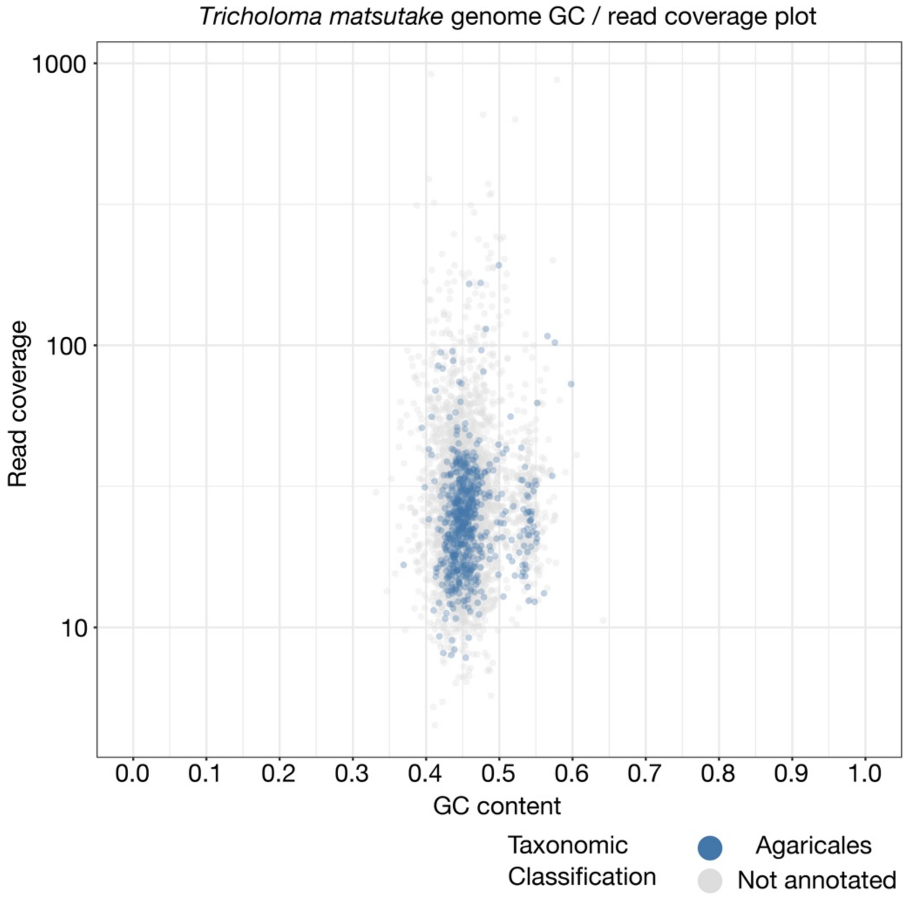

Fig B | A scatter plot of GC and read coverage of the *Tricholoma matsutake* genome. Each point indicates a scaffold marked with a color according to its taxonomic assignment (order level) which was made based on BLASTn search against the NCBI *nt* database.

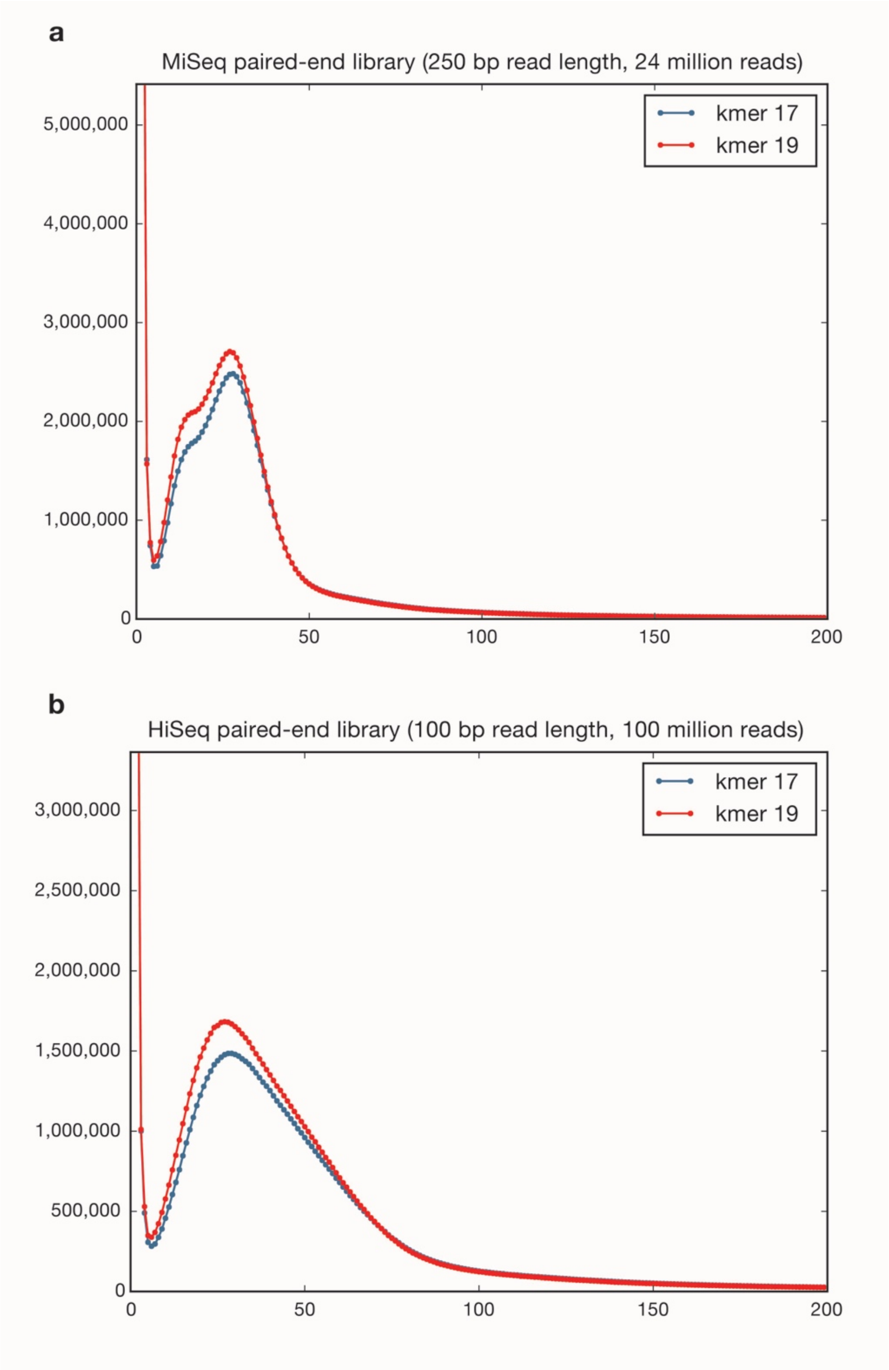

Fig C | K-mer frequency of genomic DNA sequencing. Two paired-end sequencing libraries were used.

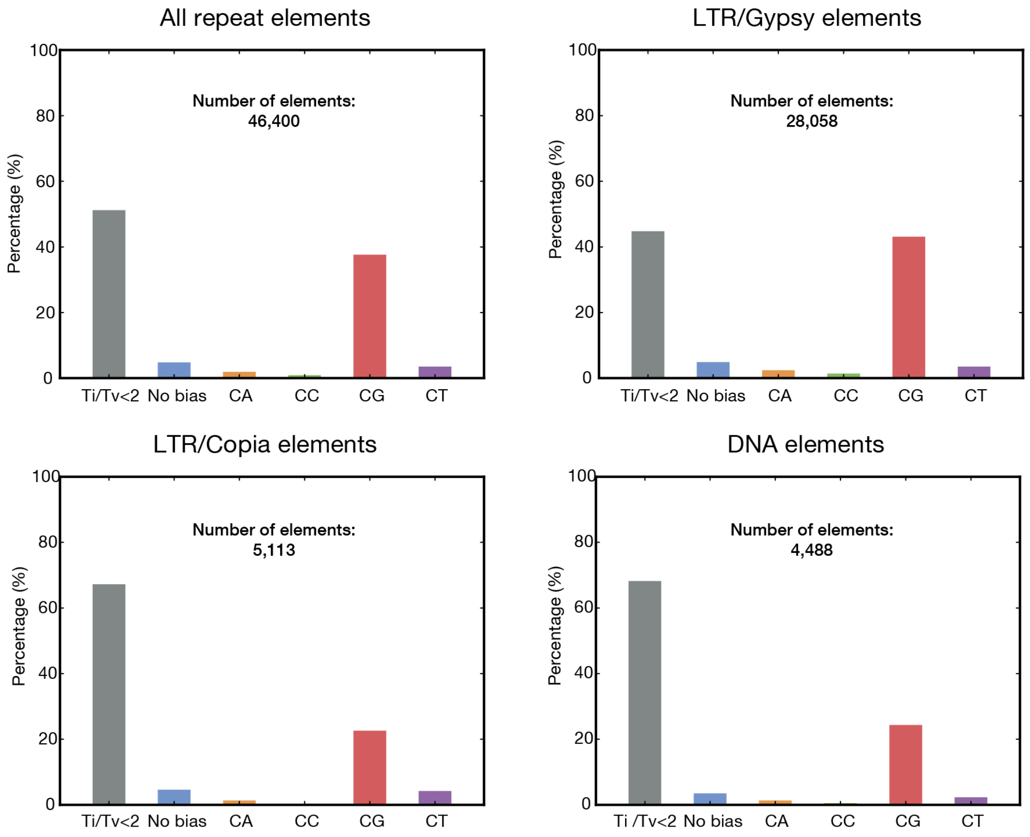

Fig D | The evidence of repeat-induced point mutation in the *T. matsutake* genome. The repeat elements showing transition/transversion ratio >2 were classified according to the dinucleotide hypermutation bias to see which dinucleotide is preferred in C to T transition during RIP process.

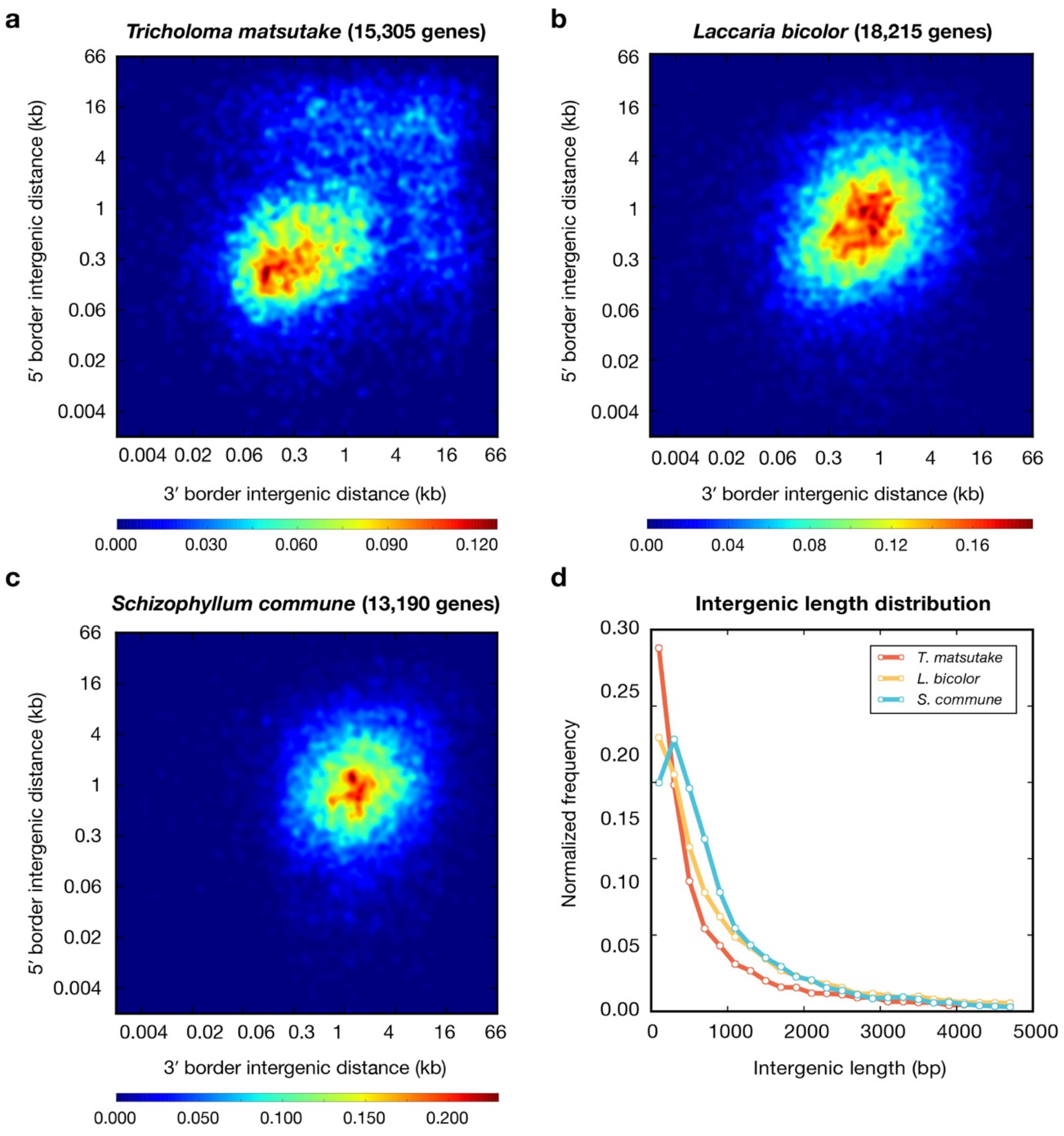

Fig E | Intergenic length distribution. a–c. GFF3 files were used to obtain the distances to neighbor genes. Both ends of genes of scaffolds were not considered because they had only one side border. Two-dimensional histograms were calculated with 1000 bins and Gaussian filter was used to smooth the heatmaps. **d.** Intergenic length distribution of three mushroom genomes. Frequency values were normalized by total gene counts of the genomes.

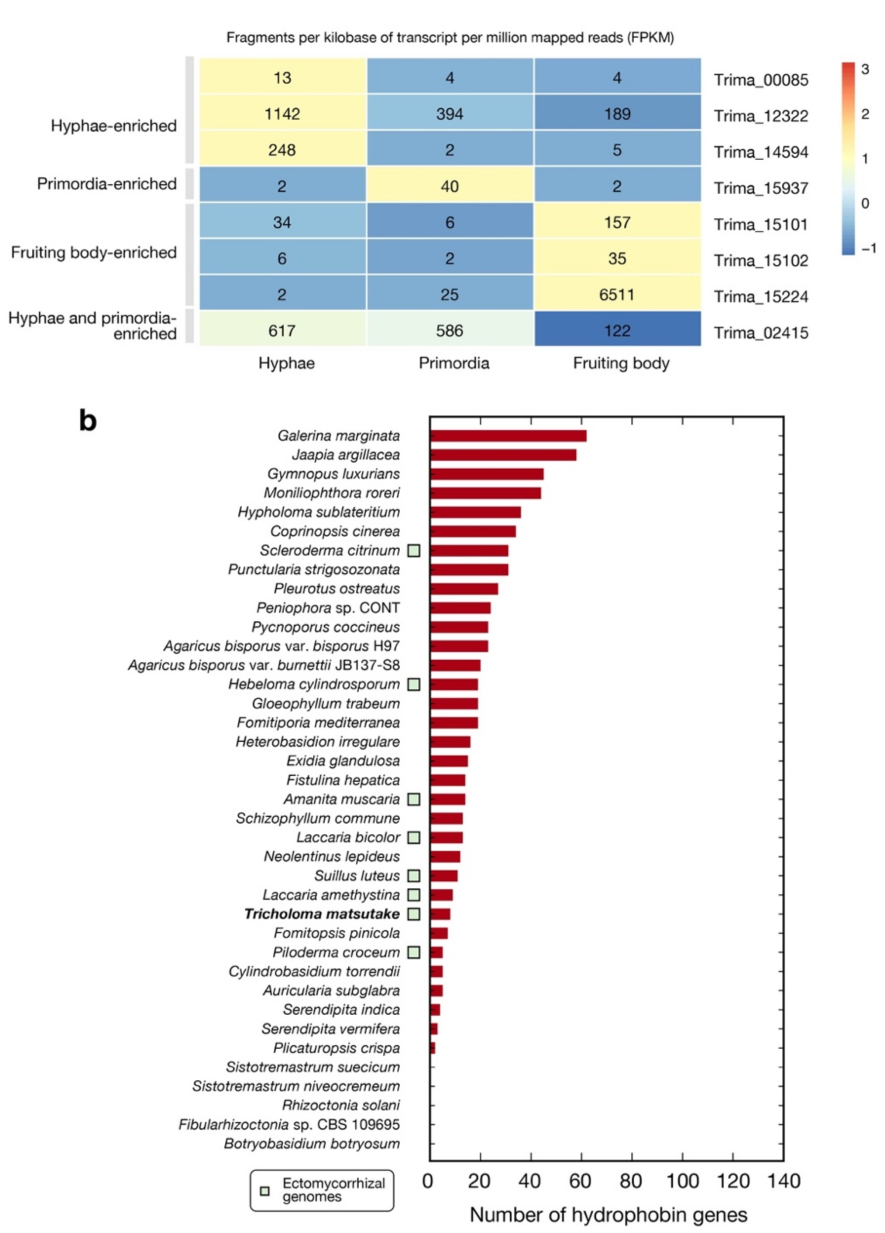

Fig F | Hydrophobin gene expression at the three developmental stages and the gene frequencies of the 37 Agaricomycetes genomes. Differentially expressed gene types were determined based on logFC (>1 or <-1) estimated by IsoDE2, based on bootstrapping of aligned reads. When two conditions were more expressed than the other, but there was no difference between them (-1<logFC<1), we assigned this gene as co-overexpressed in those two conditions. Scaled values based on row Z-scores were used to fill each cell. The genomes list is shown in Supplementary Table S3.

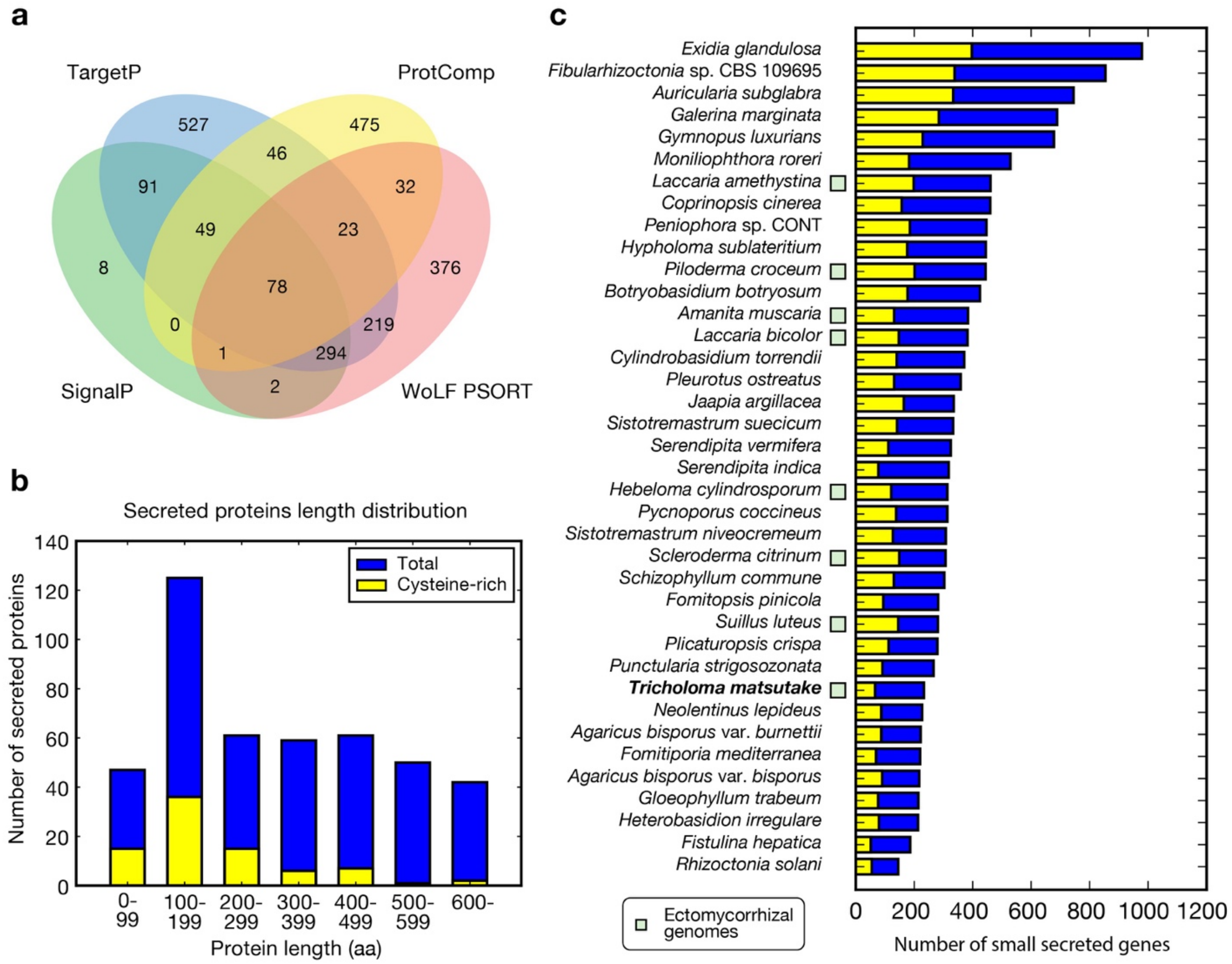

Fig G | Secreted protein genes. a. The Venn diagram of predicted secreted protein genes predicted by four different programs. **b.** Length distribution of secreted proteins of the *T. matsutake* genome. Cysteine-rich protein sequences are determined when they contain >3% cysteines in the sequence. **c.** Secreted small protein genes (<300 aa) of 38 Agaricomycetes genomes.

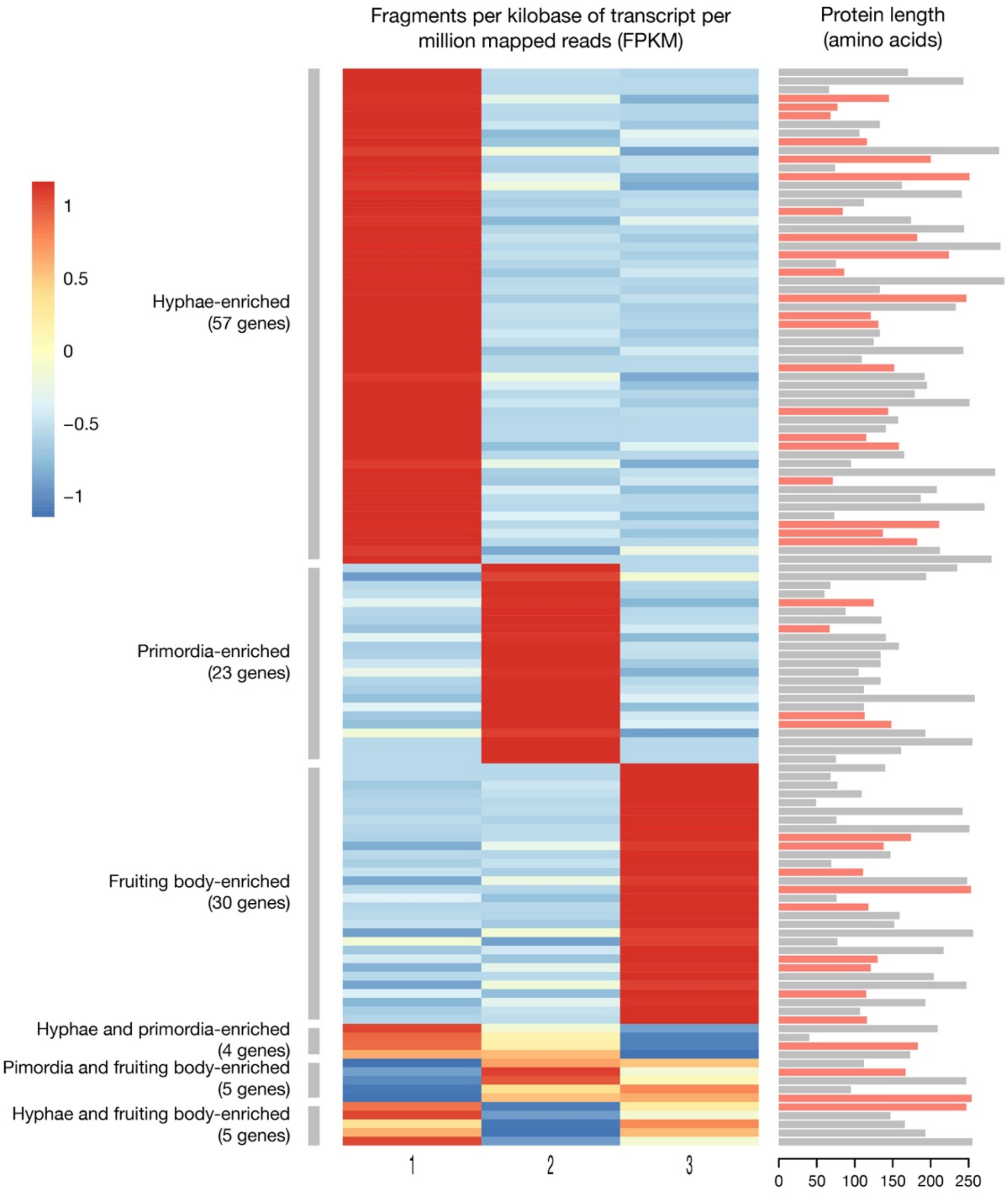

Fig H | Differentially expressed small secreted protein genes. Differentially expressed genes were determined by IsoDE2, based on bootstrapping of aligned reads. Less than 300 aa length of proteins were considered. Cysteine-rich proteins (>3% of a protein sequence) were marked with light pink color at the protein length bars. Scaled values based on row Z-scores were used to fill each cell.

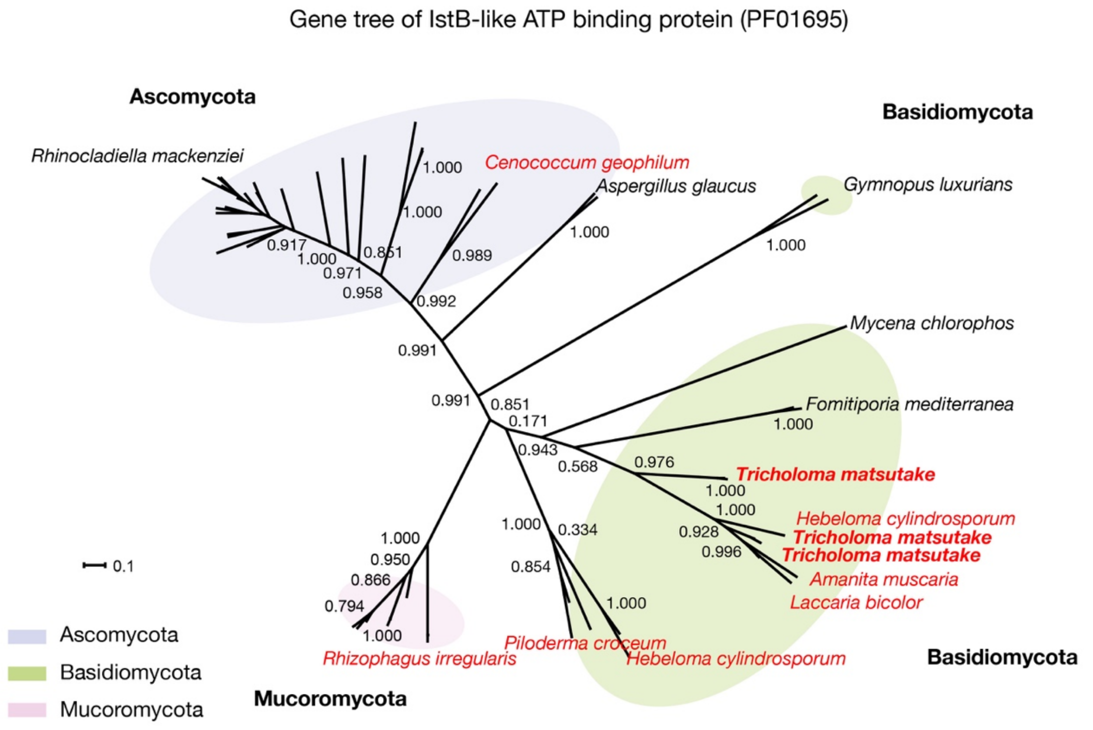

Fig I | Gene tree of IstB-like ATP binding protein. The 50 homologs were retrieved from BLAST search against the NCBI *nr* database. The gene tree was built using Mafft 7.273 for multiple sequence alignment and FastTree for tree building. The scale bar that represents the mean number of amino acid substitutions per site is shown. Local support values computed with the Shimodaira-Hasegawa test are indicated. Mycorrhizal species are marked in red color.

Gene tree of cadmium resistance transporter (PF03596)

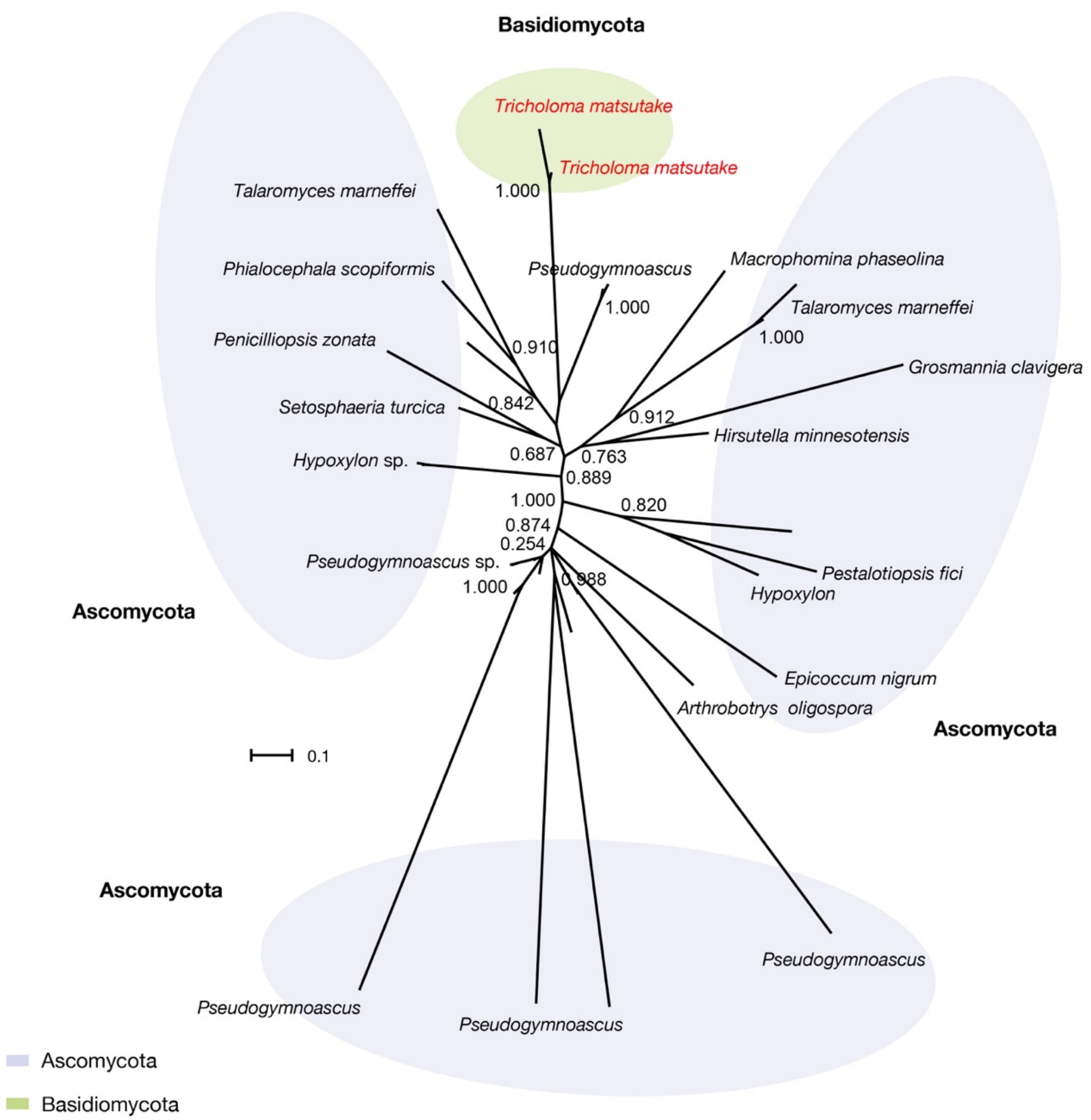

Fig J | Gene tree of cadmium resistance transporter. The 50 homologs were retrieved from BLAST search against the NCBI *nr* database. The gene tree was built using Mafft 7.273 for multiple sequence alignment and FastTree for tree building. Local support values computed with the Shimodaira-Hasegawa test are indicated. The scale bar that represents the mean number of amino acid substitutions per site is shown. *Tricholoma matsutake* is marked with red color.

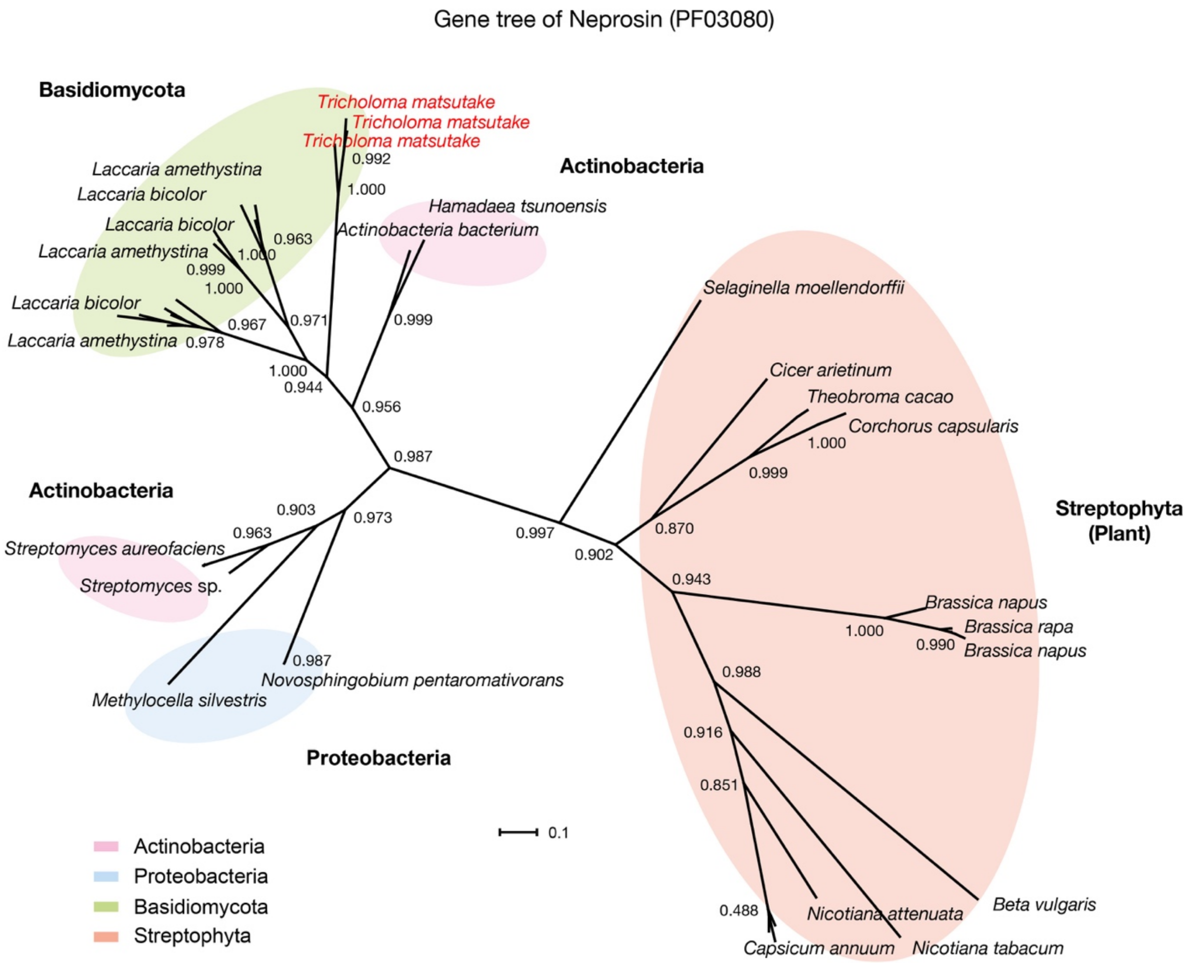

Fig K | Gene tree of neprosin. The 50 homologs were retrieved from BLAST search against the NCBI *nr* database. The gene tree was built using Mafft 7.273 for multiple sequence alignment and FastTree for tree building. Local support values computed with the Shimodaira-Hasegawa test are indicated for each branch. The scale bar that represents the mean number of amino acid substitutions per site is shown. *Tricholoma matsutake* is marked with red color.

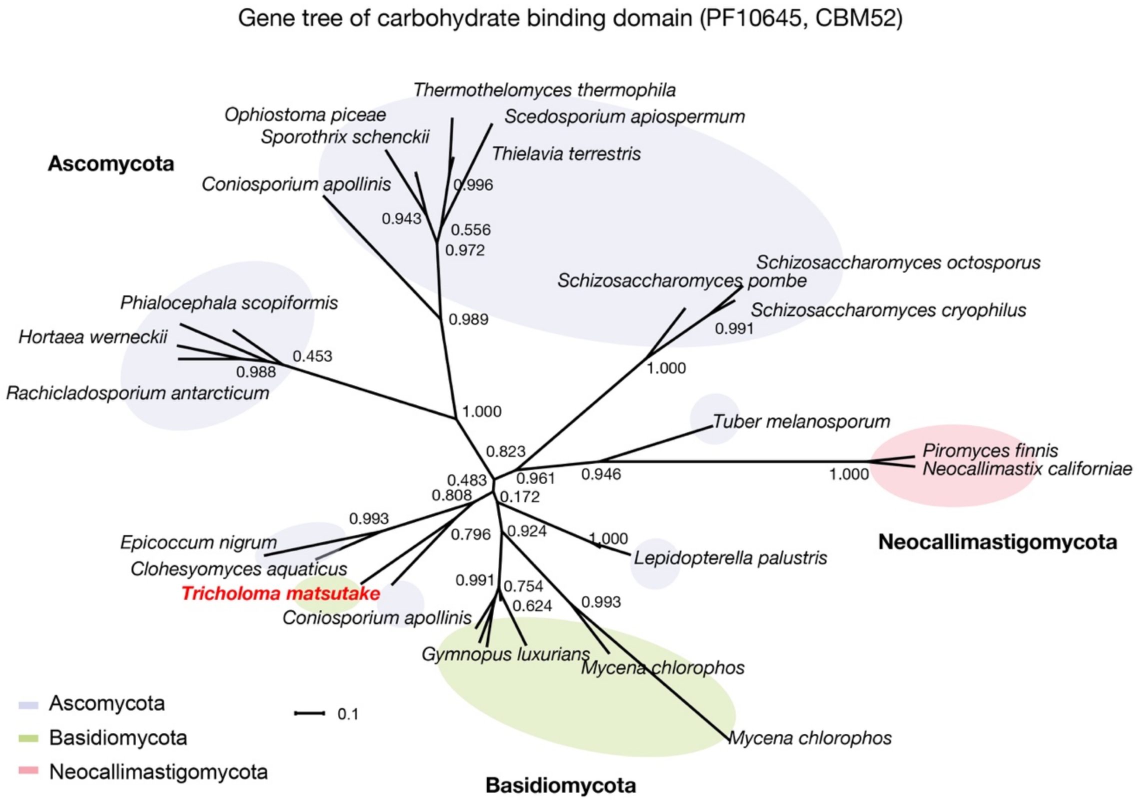

Fig L | Gene tree of carbohydrate binding domain. The 30 homologs were retrieved from BLAST search against the NCBI *nr* database. The gene tree was built using Mafft 7.273 for multiple sequence alignment and FastTree for tree building. Local support values computed with the Shimodaira-Hasegawa test are indicated for each branch. The scale bar that represents the mean number of amino acid substitutions per site is shown. *Tricholoma matsutake* is marked with red color.

Table A. Target proteins responsible for genome defense by fungi

Mechanism	Name	Description	Source organism	Length (aa)	GenBank accession
Repeat-induced point mutation	Rid/Dim2[1]	DNA methyltransferase	*Neurospora crassa*	1454	AAK49954.1
	Masc1[2]	C5-DNA methyltransferase	*Ascobolus immersus*	537	AAC49849.1
	Masc2[3]	C5-DNA methyltransferase	*Ascobolus immersus*	1356	AAC03766.1
Meiotic silencing by unpaired DNA	Sad-1[4]	RNA dependent RNA polymerase	*Neurospora crassa*	1638	EAA35012.3
	Sms-3[4]	dicer-like protein	*Neurospora crassa*	1584	EAA32662.1
	Sms-2[4]	meiotic silencing suppressor	*Neurospora crassa*	989	EAA29350.1
Quelling	Qde-1[4]	RNA-dependent RNA polymerase	*Neurospora crassa*	1402	EAA29811.1
	Dcl2[4]	dicer-like protein	*Neurospora crassa*	1396	XP_963538.3
	Qde2[5]	post-transcriptional gene silencing	*Neurospora crassa*	938	AAF43641.1

Table B. Transcription factors involved in mushroom formation

Protein name	GenBank Access	Length (aa)	Homologs in *Tricholoma matsutake*	Length (aa)	E-value	Identity (%)
Hom2	XP_003029756.1	723	Trima_14430	679	2.49e-57	33.4
Bri1	XP_003038897.1	776	Trima_08826	932	1.99e-159	39.2
Fst4	XP_003034563.1	826	Trima_15097	859	0.0	45.1
C2H2	XP_003026630.1	360	Trima_03635	486	7.59e-32	61.9
Fst3	XP_003031320.1	1120	Trima_16495	1056	0.0	64.8
Gat1	XP_003036589.1	449	Trima_00186	527	6.07e-66	39.1
Hom1	XP_003030056.1	316	Trima_12069	379	7.22e-31	75.6

Table C. The summary of genome sequencing

Platform	Insert size (bp)	Number of reads	Average read length (bp)	Total bases (bp)
HiSeq paired-end	500	99,204,510	100	10,019,655,510
HiSeq mate pair	5,000	310,061,732	100	31,316,234,932
MiSeq paired-end	250	24,712,074	250	6,165,993,276

Table D. Transposable element Pfam domains

Pfam ID	Pfam description
PF00075	RNase H
PF00078	Reverse transcriptase (RNA-dependent DNA polymerase)
PF00665	Integrase core domain
PF02925	Bacteriophage scaffolding protein D
PF02992	Transposase family tnp2
PF03184	DDE superfamily endonuclease
PF03221	Tc5 transposase DNA-binding domain
PF03732	Retrotransposon gag protein
PF04687	Microvirus H protein (pilot protein)
PF05699	hAT family C-terminal dimerisation region
PF05840	Bacteriophage replication gene A protein (GPA)
PF05970	PIF1-like helicase
PF07727	Reverse transcriptase (RNA-dependent DNA polymerase)
PF08283	Geminivirus rep protein central domain
PF08284	Retroviral aspartyl protease
PF10551	MULE transposase domain
PF13358	DDE superfamily endonuclease
PF13359	DDE superfamily endonuclease
PF13456	Reverse transcriptase-like
PF13837	Myb/SANT-like DNA-binding domain
PF13976	GAG-pre-integrase domain
PF14214	Helitron helicase-like domain at N-terminus
PF14223	gag-polypeptide of LTR copia-type
PF14529	Endonuclease-reverse transcriptase

Table E. Genomes used for comparative analysis

Species	Order	GenBank ID	Ecotype	Number of genes	Genome Size (Mbp)
Agaricus bisporus var. *bisporus* H97	Agaricales	GCF_000300575.1	Saprotroph	10,448	30.2
Agaricus bisporus var. *burnettii* JB137-S8	Agaricales	GCF_000300555.1	Saprotroph	11,278	32.6
Amanita muscaria Koide BX008	Agaricales	GCA_000827485.1	Ectomycorrhizal	18,093	40.7
Auricularia subglabra TFB-10046 SS5	Auriculariales	GCF_000265015.1	White-rot	23,555	74.9
Botryobasidium botryosum FD-172 SS1	Cantharellales	GCA_000697705.1	White-rot	16,502	46.7
Coprinopsis cinerea okayama7#130	Agaricales	GCF_000182895.1	Saprotroph	13,356	36.2
Cylindrobasidium torrendii FP15055 ss-10	Agaricales	GCA_000934385.1	White-rot	13,936	31.6
Exidia glandulosa HHB12029	Auriculariales	GCA_001632375.1	Saprotroph	26,690	78.2
Fibularhizoctonia sp. CBS 109695	Atheliales	GCA_001630335.1	Saprotroph	32,854	95.1
Fistulina hepatica ATCC 64428	Agaricales	GCA_000934395.1	Brown-rot	11,244	33.8
Fomitiporia mediterranea MF3/22	Hymenochaetales	GCF_000271605.1	White-rot	11,338	63.4
Fomitopsis pinicola FP-58527 SS1	Polyporales	GCA_000344655.2	Brown-rot	13,852	41.6
Galerina marginata CBS 339.88	Agaricales	GCA_000697645.1	White-rot	21,391	59.4
Gloeophyllum trabeum ATCC 11539	Gloeophyllales	GCF_000344685.1	Brown-rot	11,755	37.2
Gymnopus luxurians FD-317 M1	Agaricales	GCA_000827265.1	Saprotroph	22,046	66.3
Hebeloma cylindrosporum h7	Agaricales	GCA_000827355.1	Ectomycorrhizal	15,376	38.2
Heterobasidion irregulare TC 32-1	Russulales	GCF_000320585.1	Plant pathogen	13,275	33.6
Hypholoma sublateritium FD-334 SS-4	Agaricales	GCA_000827495.1	White-rot	17,771	48
Jaapia argillacea MUCL 33604	Jaapiales	GCA_000697665.1	White-rot	16,375	45
Laccaria amethystina LaAM-08-1	Agaricales	GCA_000827195.1	Ectomycorrhizal	21,033	52.2
Laccaria bicolor S238N-H82	Agaricales	GCF_000143565.1	Ectomycorrhizal	18,215	64.9
Moniliophthora roreri MCA 2997	Agaricales	GCF_000488995.1	Plant pathogen	17,910	52.2
Neolentinus lepideus HHB14362 ss-1	Gloeophyllales	GCA_001632425.1	Saprotroph	13,157	35.6
Peniophora sp. CONT	Russulales	GCA_001632445.1	White-rot	18,945	46
Piloderma croceum F 1598	Atheliales	GCA_000827315.1	Ectomycorrhizal	21,524	59.3
Pleurotus ostreatus PC15	Agaricales	GCA_000697685.1	White-rot	12,296	34.3
Plicaturopsis crispa FD-325 SS-3	Amylocorticiales	GCA_000827205.1	White-rot	13,617	34.5
Punctularia strigosozonata HHB-11173 SS5	Corticiales	GCF_000264995.1	White-rot	11,540	34.2
Pycnoporus coccineus BRFM310	Polyporales	GCA_002092935.1	White-rot	12,693	32.8
Rhizoctonia solani AG-1 IA	Cantharellales	GCA_000334115.1	Plant pathogen	10,489	36.9
Schizophyllum commune H4-8	Agaricales	GCF_000143185.1	White-rot	13,194	38.5
Scleroderma citrinum Foug A	Boletales	GCA_000827425.1	Ectomycorrhizal	20,995	56.1
Serendipita indica DSM 11827	Sebacinales	GCA_000313545.1	Endophyte	11,791	25
Serendipita vermifera MAFF 305830	Sebacinales	GCA_000827415.1	Orchid mycorrhizal	15,245	38.1

Sistotremastrum niveocremeum HHB9708	Trechisporales	GCA_001630475.1	Saprotroph	13,076	35.4
Sistotremastrum suecicum HHB10207 ss-3	Trechisporales	GCA_001632355.1	Saprotroph	13,654	33.9
Suillus luteus UH-Slu-Lm8-n1	Boletales	GCA_000827255.1	Ectomycorrhizal	18,303	37

Table F. Total bases and read counts for the three developmental stages of *Tricholoma matsutake*

Development stage	Total bases	Read count	N (%)	GC (%)	Q20 (%)	Q30 (%)
Hyphae	10,954,625,640	108,461,640	0.004	49.58	94.12	86.92
Primordia	13,424,362,278	132,914,478	0.004	49.85	93.95	86.82
Fruiting Body	12,872,445,152	127,449,952	0.004	49.79	94.34	87.42

Table G. Repeat families predicted by RepeatModeler

Repeat family	Number of families	Average length (bp)
LTR	289	1832
DNA	130	2398
LINE	39	2228
RC	22	2103
Simple_repeat	14	756
buffer	2	2098
Unknown	1879	676

Supplementary references

1 Kouzminova, E. & Selker, E. U. dim-2 encodes a DNA methyltransferase responsible for all known cytosine methylation in Neurospora. *The EMBO journal* **20**, 4309-4323, doi:10.1093/emboj/20.15.4309 (2001).

2 Malagnac, F. *et al.* A gene essential for de novo methylation and development in Ascobolus reveals a novel type of eukaryotic DNA methyltransferase structure. *Cell* **91**, 281-290 (1997).

3 Chernov, A. V., Vollmayr, P., Walter, J. & Trautner, T. A. Masc2, a C5-DNA-methyltransferase from Ascobolus immersus with similarity to methyltransferases of higher organisms. *Biological chemistry* **378**, 1467-1473 (1997).

4 Galagan, J. E. *et al.* The genome sequence of the filamentous fungus Neurospora crassa. *Nature* **422**, 859-868, doi:10.1038/nature01554 (2003).

5 Catalanotto, C., Azzalin, G., Macino, G. & Cogoni, C. Gene silencing in worms and fungi. *Nature* **404**, 245, doi:10.1038/35005169 (2000).
